# Ligand impact on reactive oxygen species generation of Au_10_ and Au_25_ nanoclusters upon one- and two-photon excitation

**DOI:** 10.1038/s42004-023-00895-5

**Published:** 2023-05-22

**Authors:** Hussein Fakhouri, Martina Perić Bakulić, Issan Zhang, Hao Yuan, Dipankar Bain, Fabien Rondepierre, Pierre-François Brevet, Željka Sanader Maršić, Rodolphe Antoine, Vlasta Bonačić-Koutecký, Dusica Maysinger

**Affiliations:** 1 Institut Lumière Matière, University of Lyon, Université Claude Bernard Lyon 1, CNRS, F-69622 Lyon, France; 2 Center of Excellence for Science and Technology, Integration of Mediterranean Region (STIM), Faculty of Science, University of Split, Ruđera Boškovića 33, 21000 Split, Croatia; 3 Faculty of Chemistry and Technology, University of Split, Rudera Boskovica 35, 21000 Split, Croatia; 4 Department of Pharmacology and Therapeutics, McGill University, 3655 Promenade Sir-William-Osler, H3G 1Y6 Montreal, Canada; 5 Faculty of Science, University of Split, Rudera Boskovica 33, 21000 Split, Croatia; 6 Interdisciplinary Center for Advanced Science and Technology (ICAST), University of Split, Meštrovićevo šetalište 45, 21000 Split, Croatia; 7 Chemistry Department, Humboldt University of Berlin, Brook-Taylor-Strasse 2, 12489 Berlin, Germany

**Keywords:** Photobiology, Nanomedicine, Nanoparticles

## Abstract

In photodynamic therapy (PDT), light-sensitive photosensitizers produce reactive oxygen species (ROS) after irradiation in the presence of oxygen. Atomically-precise thiolate-protected gold nanoclusters are molecule-like nanostructures with discrete energy levels presenting long lifetimes, surface biofunctionality, and strong near-infrared excitation ideal for ROS generation in PDT. We directly compare thiolate-gold macromolecular complexes (Au_10_) and atomically-precise gold nanoclusters (Au_25_), and investigate the influence of ligands on their photoexcitation. With the ability of atomically-precise nanochemistry, we produce Au_10_SG_10_, Au_10_AcCys_10_, Au_25_SG_18_, and Au_25_AcCys_18_ (SG: glutathione; AcCys: N-acetyl-cysteine) fully characterized by high-resolution mass spectrometry. Our theoretical investigation reveals key factors (energetics of excited states and structural influence of surface ligands) and their relative importance in singlet oxygen formation upon one- and two-photon excitation. Finally, we explore ROS generation by gold nanoclusters in living cells with one- and two-photon excitation. Our study presents in-depth analyses of events within gold nanoclusters when photo-excited both in the linear and nonlinear optical regimes, and possible biological consequences in cells.

## Abstract (web summary)

Atomically precise thiolate-protected gold nanoclusters present strong near-infrared excitation, long lifetimes, and surface biofunctionality, making them ideal candidates as photosensitizers in photodynamic therapy. Here, the authors evaluate the influence of the ligands and metal core on the efficiency of photoexcited Au_10_ and Au_25_ clusters to produce reactive oxygen species, as well as possible biological consequences in living cells.

## Introduction

**Fig. 1:**
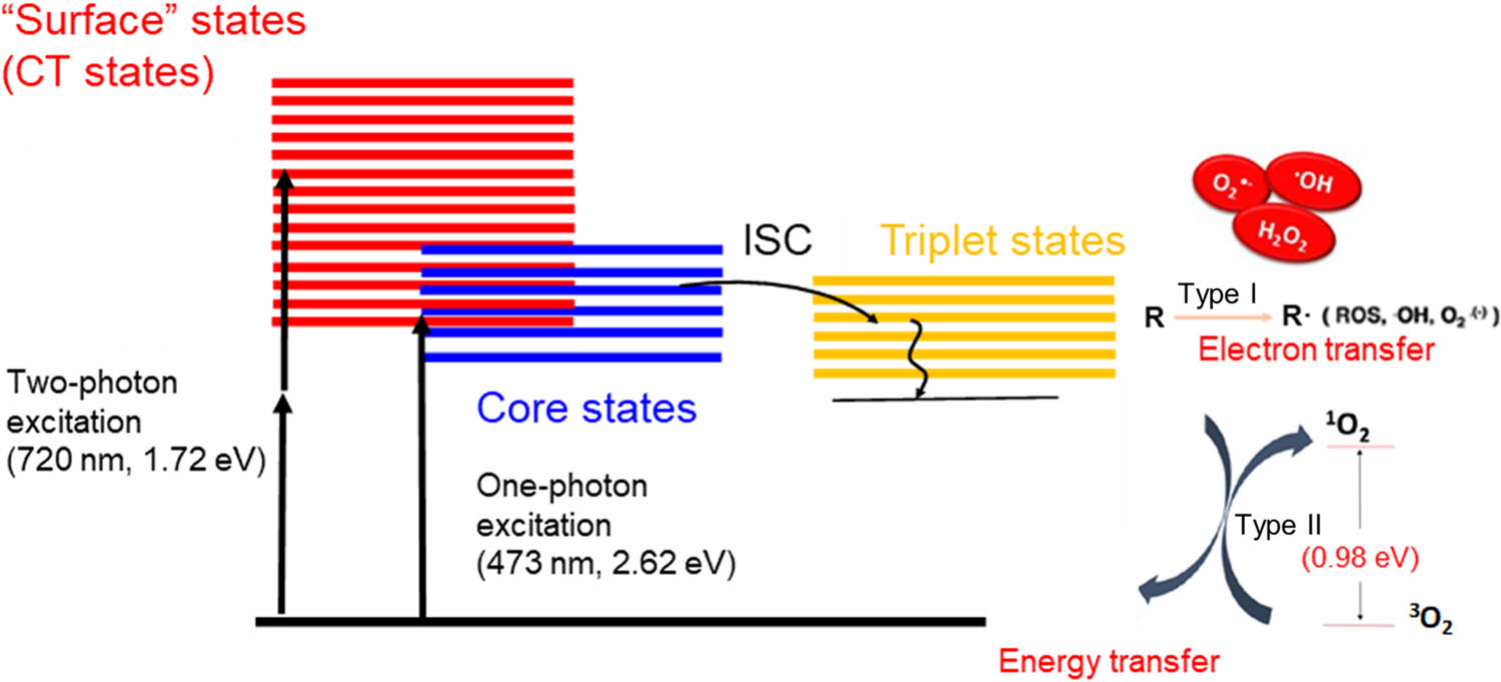
Type I and type II mechanism of ROS generation using photoexcited gold nanoclusters (upon one- and two-photon excitation). CT charge transfer, ISC intersystem crossing, ${{{{{{{\rm{O}}}}}}}_{2}}^{\cdot -}$, superoxide anion, •OH hydroxyl radical, H_2_O_2_ hydrogen peroxide, ^3^O_2_ triplet state oxygen (molecular oxygen), ^1^O_2_ singlet oxygen.

Photodynamic therapy (PDT) is a powerful therapeutic method using a light-sensitive compound or structure, commonly named a photosensitizer to produce reactive oxygen species (ROS)^1^ after irradiation with light in the presence of oxygen^2^. A photosensitizer molecule is usually irradiated by visible or near-infrared (NIR) light. A photosensitizer absorbs the light and is excited to its singlet state. The excited state electrons undergo intersystem crossing to a lower energy, but longer-lived triplet state, from which ROS or reactive molecular transients are generated (Fig. 1). The photochemical reactions proceed via a type I (by electron transfer) or type II (by energy transfer) mechanism and require close proximity between the photosensitizer and molecular oxygen. The photosensitizer should possess high light absorption coefficients, ideally at long wavelength radiations (red or near-infrared), long lifetime (to allow high intersystem crossing efficiencies), and good biocompatibility (in the absence of light). Many structures, ranging from single organic-based molecules, thiolate-metal complexes^3^ to tailor-made nanomaterials, have been used as photosensitizers^4^. Atomically precise thiolate (SR)-protected gold nanoclusters (Au_n_(SR)_m_) are molecule-like nanostructures^5,6^ presenting long lifetimes, surface biofunctionality, and strong NIR excitation that are thus ideal candidates for generating ROS—in particular singlet oxygen (^1^O_2_). It was shown by pioneering studies that Au_25_(SR)_18_ excited either by light^7,8^ or by ultrasound^9^ can donate enough energy to convert ^3^O_2_ into ^1^O_2_. In addition, atomically precise gold nanoclusters (mainly protected by proteins) have been recently proposed as Type-I-Type-II sensitizers for potential use in PDT either using one-photon red or NIR light^10–14^, or two-photon excitation^8,15^.

A few experimental studies have tried to address size effects either between different Au_n_SR_m_ atomically precise nanoclusters^16^ or between molecule-like clusters and plasmonic gold nanoparticles^8^. However, to our knowledge, no direct comparison between thiolate-gold macromolecular complexes and atomically precise gold nanoclusters has been explored. Thus, Au_10_ (thiolate-gold macromolecular complexes) and Au_25_ (atomically precise gold nanoclusters) were chosen to highlight how discrete energy levels and the nature of excited states may influence the photosensitizing abilities of atomically precise gold clusters to produce ROS (in particular ^1^O_2_). Indeed, as illustrated in Fig. 1, an efficient photosensitizer for ^1^O_2_ production requires a high triplet-state yield with a triplet-state energy higher than the energy of ^1^O_2_ (0.98 eV) for efficient energy transfer to ^3^O_2_. From this viewpoint, the optical gap of thiolate-protected Au_25_(SR)_18_ nanoclusters is slightly higher than 1 eV^17,18^, and due to the long-lifetime, the triplet-state efficiency is high. This is mainly due to the strong interaction between core states in the gold core and the surface states at the Au-S interface. In contrast, the optical gap of Au_10_SR_10_ is much higher, at ~2.6–2.7 eV^17,19,20^, and therefore it can also donate enough energy to form ^1^O_2_. However, as opposed to Au_25_(SR)_18_ containing 8 confined electrons in the gold core, Au_10_(SR)_10_ presents a catenane structure with zero confined electrons^19–22^. Aurophilic Au···Au subunits with neighboring sulfur atoms in catenane structures of Au_10_ nanoclusters play a key role in the photophysical processes^19^.

In addition to the effect of molecular-like properties of ultrasmall nanoclusters, our aim is to evaluate the influence of ligands on the efficiency of photoexcited gold clusters to produce ROS. Wu and Jin did a seminal work demonstrating that ligands play a key role on the photoluminescence efficiencies of thiolate-protected nanoclusters^23^. Since ROS generation involves subtle photophysical relaxation processes in excited states, it is thus postulated that ligands should also play a role in the ROS generation of photoexcited nanoclusters. However, very few studies explore the possible impact of ligands, mainly focusing on Au_25_ capped with thiolate molecules (captopril, 4-mercaptobenzoic acid), peptides (glutathione) or proteins (albumin)^16,24^.

In this work, with the ability of atomically precise nanochemistry, our aim is to produce Au_10_SG_10_, Au_10_AcCys_10_, Au_25_SG_18_ and Au_25_AcCys_18_, (SG: glutathione; AcCys: N-acetyl-cysteine) which are fully characterized by high-resolution mass spectrometry^25^. Reactive oxygen species generation studies were conducted on Au_10_ and Au_25_ nanoclusters with different mode of photoexcitation (either by one-photon excitation with visible light or by two-photon excitation with NIR light). The effectiveness of such nanoclusters as photosensitizers has been evaluated in solution with an indirect singlet oxygen detection method under visible excitation using a continuous wave laser emitting at 473 nm and under NIR excitation using a femtosecond laser emitting at 780 nm and 720 nm. In such scenario, only the first excited states of nanoclusters are supposed to be involved in the photoexcitation process. Singlet oxygen formation is a low energy process (0.98 eV), the gap between singlet (S) and triplet (T) states as well as the energy of the lowest T_1_ states are key factors to evaluate the possible difference between Au_10_ and Au_25_ to produce single oxygen. Therefore, in parallel to this experimental investigation, the excited states involved in the photoexcitation and de-excitation processes are discussed based on results of time-dependent density functional theory (TDDFT) method. In order to address the possible role of surface ligands on the efficiency to produce singlet oxygen, such theoretical modeling was conducted by taking into account fully explicit ligands (glutathione and N-acetyl-cysteine) on Au_10_ and Au_25_ nanoclusters. The choice of ligands as protecting agents for gold clusters was mainly driven by their biocompatibility and their similarity from a biological point of view. Indeed, acetyl-cysteine serves as a precursor of glutathione biosynthesis^26^. Both ligands are endogenous compounds in cells and as such do not cause any toxicity under low micromolar concentrations, even when bound to gold nanoclusters. Our recent study provided some insights into the impact of Au_10_ nanoclusters on human microglia and interaction with high mobility group box 1 (HMGB1)^27^.

This joint experimental-theoretical investigation will allow to gain better insight into key factors (energetics of excited states and structural influence of surface ligands), as well as their relative importance involved in singlet oxygen formation upon photoexcitation in the visible and NIR range. In order to open this joint experimental-theoretical investigation for application-oriented perspectives, we carried out live cell imaging to explore the ability of Au_10_ and Au_25_ nanoclusters to generate ROS in cells stimulated by one- and two-photon excitation. We studied human microglia following treatments with Au_10_ and Au_25_ nanoclusters protected with two ligands (glutathione and acetyl-cysteine). In addition, our study presented herein provides in-depth analyses of events taking place within gold nanoclusters when photoexcited and their possible biological consequences in living cells.

## Results

### Singlet oxygen generation in metal nanoclusters excited by visible (one-photon excitation) and IR (two-photon excitation) light in solution

The indirect method was used to quantify singlet oxygen generation by photoexcited nanoclusters in solution. 1,3-diphenylisobenzofuran (DPBF) is known to be highly reactive toward singlet oxygen, forming endoperoxides (with different absorption properties, in particular at 412 nm) that can be used as optical probe^28^. The nanoclusters and DPBF can be excited simultaneously to generate and detect singlet oxygen. The generation of singlet oxygen was triggered by excitation of the nanoclusters with a continuous wave laser emitting at 473 nm with different times of exposure. The change in the absorption of DPBF was monitored over time at 412 nm^8^. The rate of ^1^O_2_ generation was obtained by the initial DPBF concentration change over time (Δ[DPBF]_0–15 min_/Δt) divided by the concentration of the nanoclusters. The rate of ^1^O_2_ generation is presented in Table 1 for all four gold nanoclusters. The atomic precision of as-synthesized gold nanoclusters was examined by high-resolution mass spectrometry (Supplementary Fig. 1) while the feature absorption bands were verified by UV–vis absorption spectra (Supplementary Fig. 2). Absorption spectra and change in the absorption of DPBF monitored over time are presented in the Supplementary Fig. 3. Au_25_ has better efficiency to produce ^1^O_2_ in solution than Au_10_. For a given cluster size, acetyl-cysteine further enhances ROS generation efficiency as compared to glutathione (Table 1). To demonstrate the efficiency of gold nanoclusters for ^1^O_2_ production, we compared ^1^O_2_ production by gold nanoclusters to that of the conventional dye photosensitizer new methylene blue (NMB)^7^. A superior ^1^O_2_ production efficiency was observed for Au_25_AcCys_18_ compared to NMB (Table 1). Of note, a superior ^1^O_2_ production efficiency for Au_25_Cap_t18_ clusters compared to that of NMB was already reported^7^. Clearly at 473 nm, Au_10_ is a weakly absorbing species as compared to Au_25_. This is even more dramatic in the nonlinear optical (NLO) regime upon excitation close to 800 nm (see reported two-photon absorption cross sections for Au_10_ and Au_25_)^19,29,30^. And therefore, the ^1^O_2_ generation rate also depends on the absorbance of nanoclusters at the used laser light. We thus provide as Supplementary Table 1, the normalized ^1^O_2_ generation rate of gold nanoclusters and NMB by absorbance at 473 nm. And clearly Au_10_ become more efficient than Au_25_ taking into account their absorbance at 473 nm. Photoluminescence lifetimes of the four gold nanoclusters was measured to reveal the electron states of these nanoclusters (Supplementary Fig. 4).

**Table 1:** Singlet oxygen generation rate of metal nanoclusters and dye photosensitizer under continuous wave 473 nm irradiation.

Photosensitizer	Emission peak	Quantum Yield	Long lifetime component (ns) (relative percentage of long lifetime component vs total lifetime)	^1^O_2_ generation rate (^1^O_2_ per cluster per min) @473 nm continuous wave laser irradiation
Au_10_SG_10_	615 nm	<10^−4^ (ref. ^17^)	767 (15%)	1.07 ± 0.06
Au_10_AcCys_10_	605 nm	$<$2.5 × 10^−5^ (this work)^*^	680 (17.86%)	1.09 ± 0.18
Au_25_SG_18_	~815 nm	10^−3^ (ref. ^17^)	883 (6%)	1.86 ± 0.06
Au_25_AcCys_18_	~820 nm	1.2 × 10^−4^ (this work)^*^	812 (38%)	2.71 ± 0.20
NMB	652 nm	0.04 (ref. ^56^)	33 (100%)	2.28 ± 0.11

^*^The quantum yield of Au_25_AcCys_18_ (Au_10_AcCys_10_) was estimated using the reference method and Au_25_SG_18_ (Au_10_SG_10_) as the reference. Standard error for quantum yield is 10%.

### Theoretical study of the structural and ligand effects on singlet and triplet excited states of Au_10_ and Au_25_ nanoclusters with explicit acetyl-cysteine and glutathione ligands

The aim of the theoretical investigation is to propose ligands and size of gold nanoclusters that will allow efficient generation of singlet oxygen for potential use in photodynamic therapy. For this purpose, the explicit treatment of ligands for acetyl-cysteine and glutathione has been introduced in order to compare their influence on H-bond network.

**Fig. 2:**
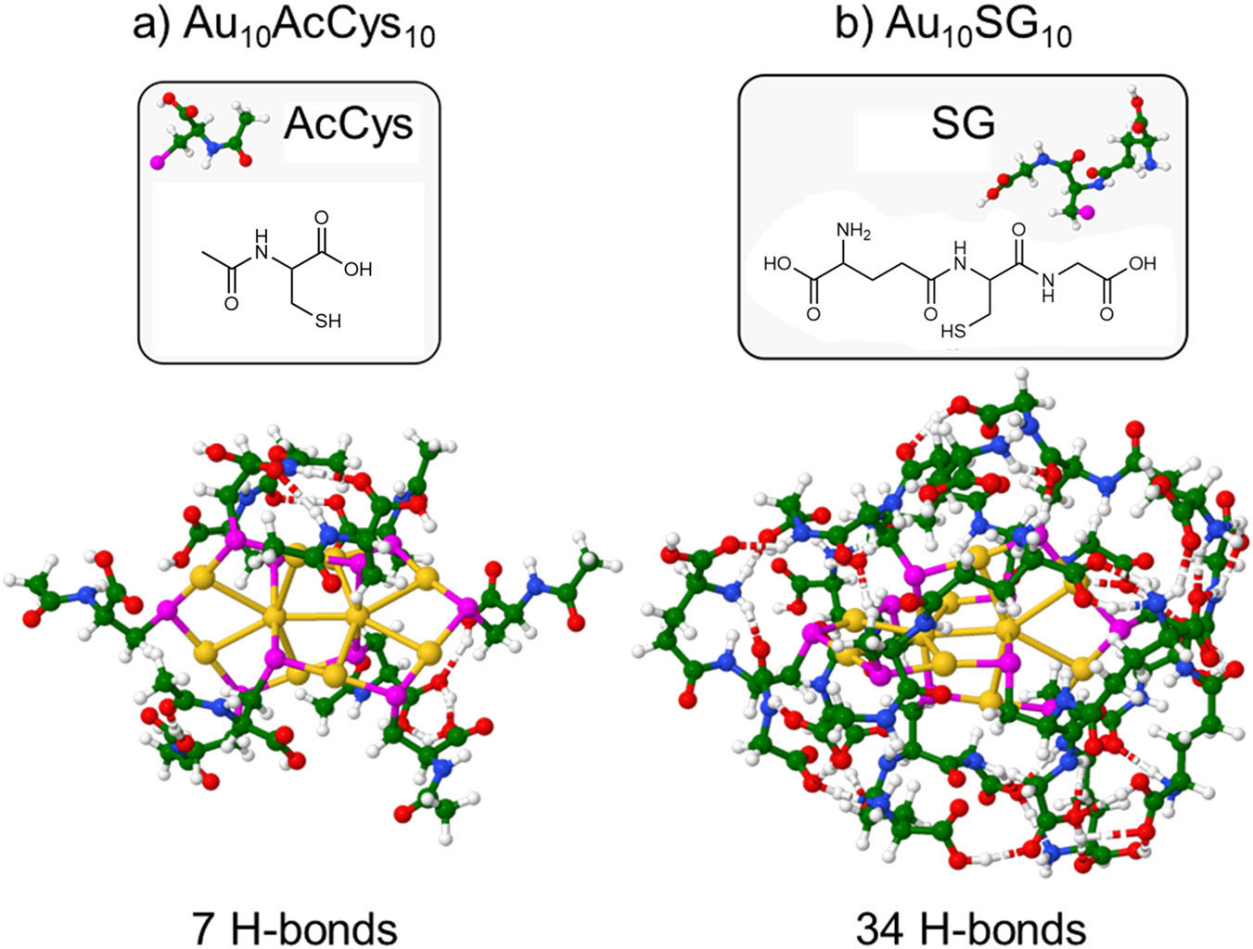
Comparison of Au_10_AcCys_10_ and Au_10_SG_10_ illustrating ligand effects by hydrogen bonding network. Structures are presented for **a** Au_10_AcCys_10_ and **b** Au_10_SG_10_. Gold atoms in structures are labeled yellow. Ligands are shown in windows. For details on structures optimization see Computational Approach. SG glutathione, AcCys N-acetyl-cysteine.

**Fig. 3:**
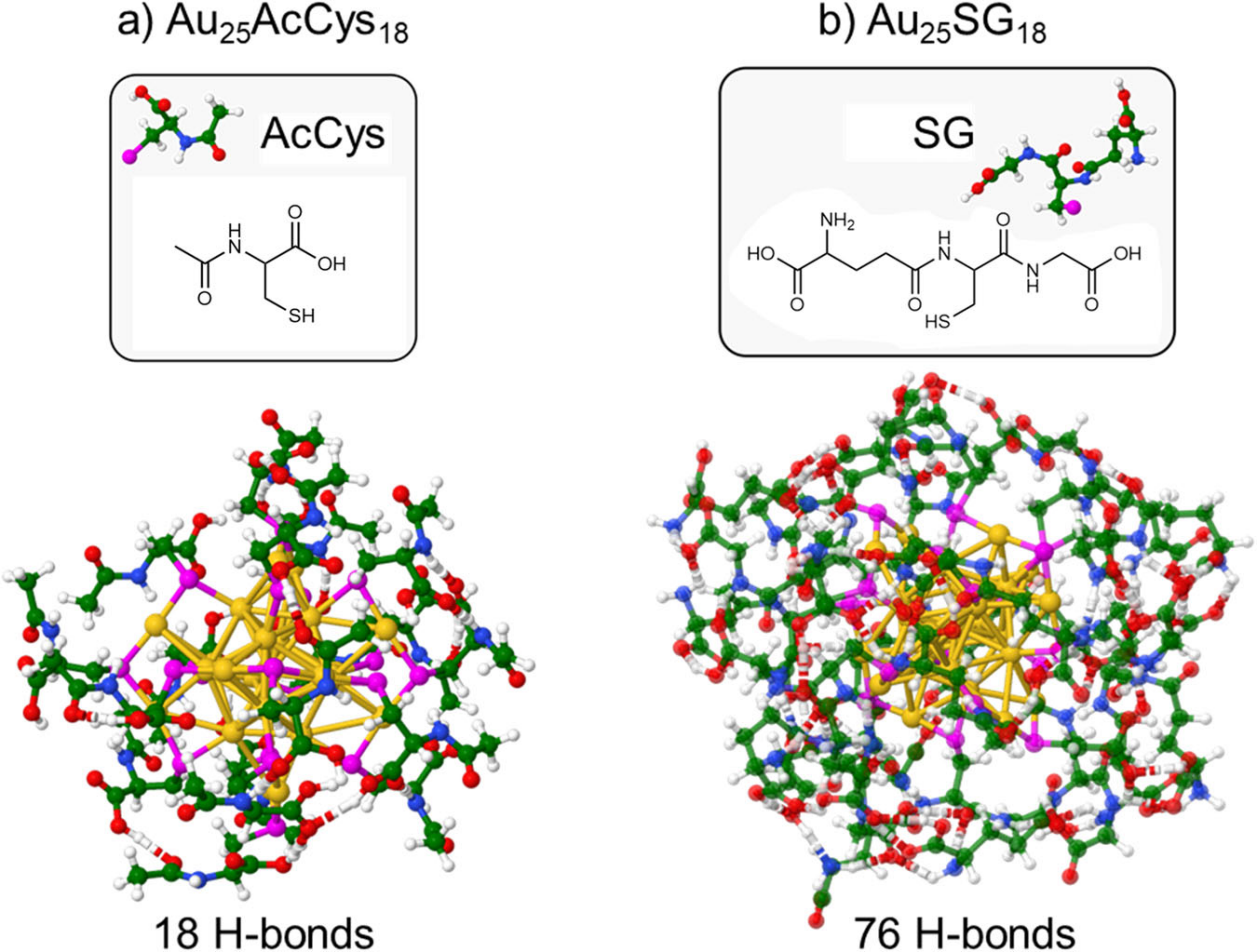
Comparison of Au_25_AcCys_18_ and Au_25_SG_18_ illustrating ligand effects by hydrogen bonding network. Structures are presented for **a** Au_25_AcCys_18_ and **b** Au_25_SG_18_. Gold atoms in structures are labeled yellow. Ligands are shown in windows. For details on structures optimization see Computational Approach. SG glutathione, AcCys N-acetyl-cysteine.

**Fig. 4:**
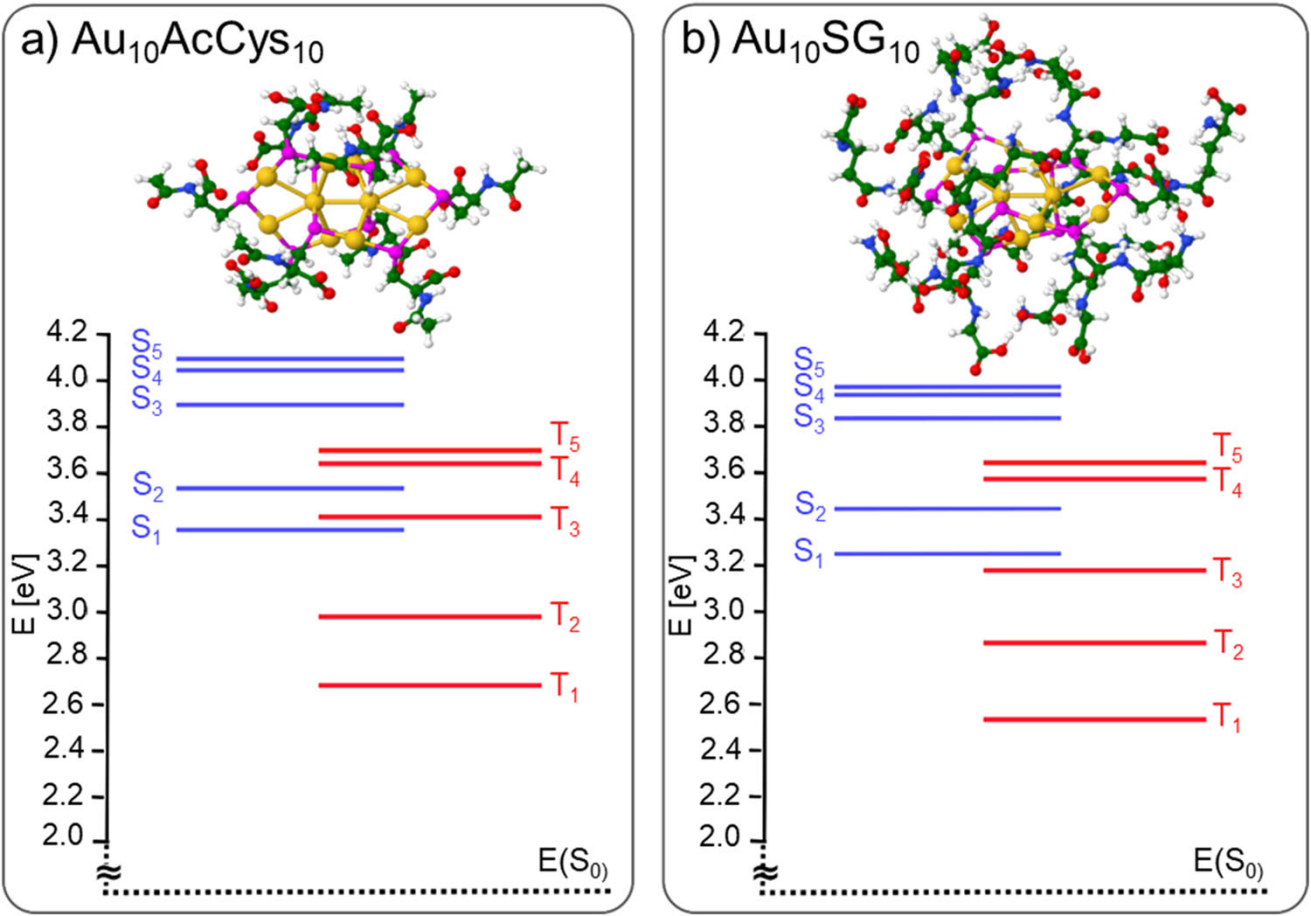
Time-dependent density functional theory (TDDFT) energies for singlets (S_n_, *n* = 1–5) and triplets (T_n_, *n* = 1–5) states. TDDFT energies are shown for **a** Au_10_AcCys_10_ and **b** Au_10_SG_10_. Singlet-triplet (E_S1_-E_T1_) energy gaps are: ΔE_S-T_ (Au_10_AcCys_10_) = 0.68 eV and ΔE_S-T_ (Au_10_SG_10_) = 0.67 eV. For details on structures optimization see Computational Approach. Gold atoms in structures are labeled yellow. Ligands are detailed in windows of Fig. 2.

**Fig. 5:**
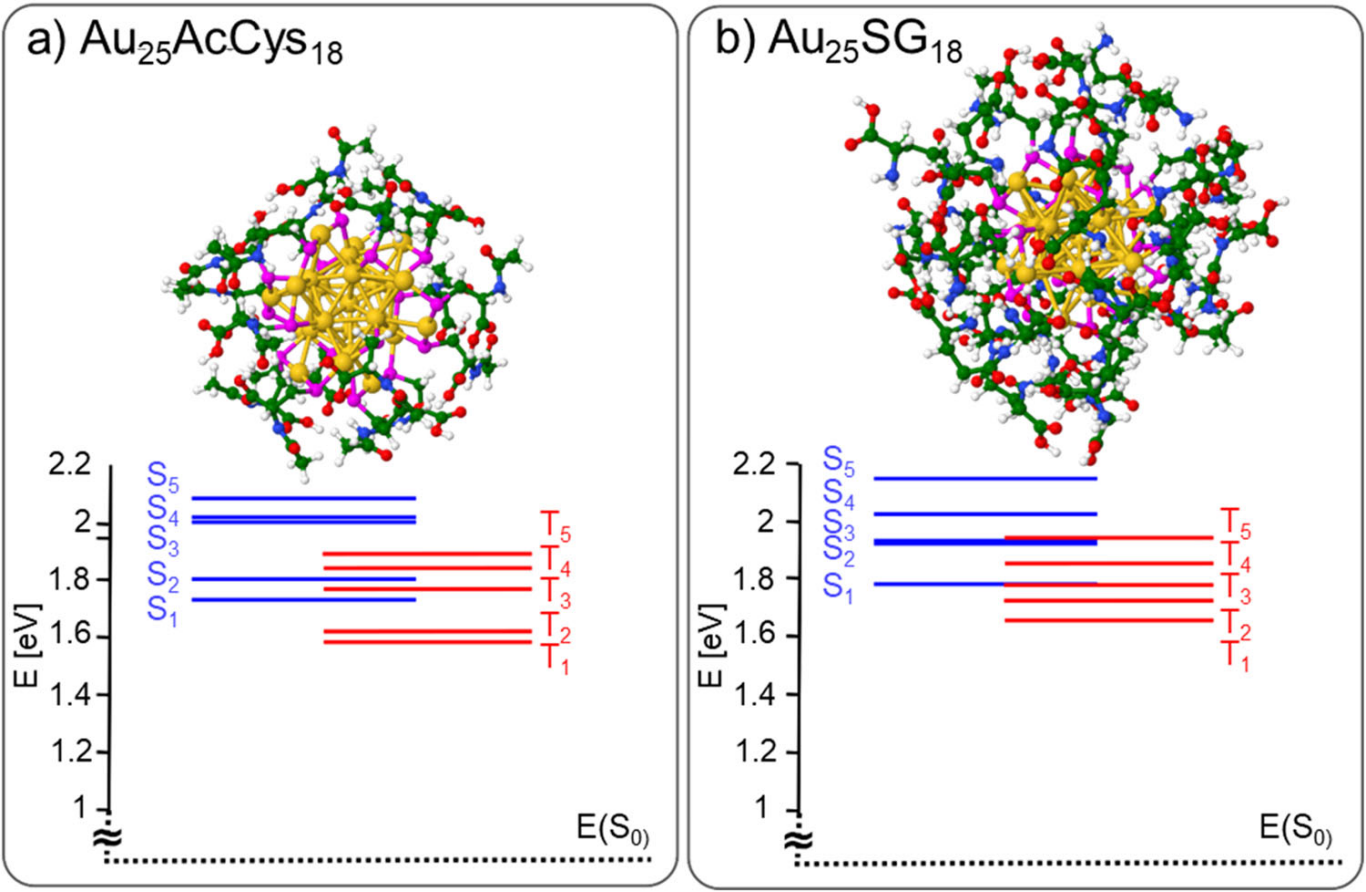
Time-dependent density functional theory (TDDFT) energies for singlets (S_n_, *n* = 1–5) and triplets (T_n_, *n* = 1–5) states. TDDFT energies are shown for **a** Au_25_AcCys_18_ and **b** Au_25_SG_18_. Singlet-triplet (E_S1_-E_T1_) energy gaps are: ΔE_S-T_ (Au_25_AcCys_18_) = 0.15 eV and ΔE_S-T_ (Au_25_SG_18_) = 0.13 eV. For details on structures optimization see Computational Approach. Gold atoms in structures are labeled yellow. Ligands are detailed in windows of Fig. 3.

Two cluster sizes, Au_10_ and Au_25_, with different structural properties and two types of ligands, AcCys and SG, are shown in Figs. 2 and 3. The Au_10_ nanocluster has a catenane type structure with interlocked ring motifs connected by Au···Au bond, whereas the Au_25_ nanocluster has a core with delocalized electrons protected by ligands. The two types of ligands are characterized as flexible (AcCys) or bulky (SG), with different flexibilities due to different H-bond networks. The greater flexibility realized by AcCys ligands allows for the transitions from singlet to triplet states. The influence of cluster size (Au_10_ vs Au_25_), along with structural properties in size regime in which each atom counts, changes drastically the relative energies of singlet-triplet states, as shown in Figs. 4 and 5 for the different ligands. This clearly evidences the advantage of Au_25_AcCys_18_ species for applications, given their close energies of singlet and triplet states and smaller number of H-bonds, promoting relaxation effects through intersystem crossing.

In the case of Au_10_AcCys_10_ and Au_10_SG_10_, excitations within the first three singlet and triplet states involve transitions between occupied and unoccupied molecular orbitals all localized at catenane bonds of interlocked ring motifs, as shown in the Supplementary Fig. 5a, b. The influence of ligands is negligible. Excitations within the first three singlet and triplet states of Au_25_AcCys_18_ and Au_25_SG_18_ species involve molecular orbitals of the gold core with delocalized electrons (Supplementary Fig. 5c, d). The influence of ligands on the relative energies of lowest singlet and triplet states is negligible since they do not participate in excitations. In contrast, size effect is pronounced due to the different structural properties.

### The ability of Au_10_ and Au_25_ nanoclusters to generate reactive oxygen species in cells

**Fig. 6:**
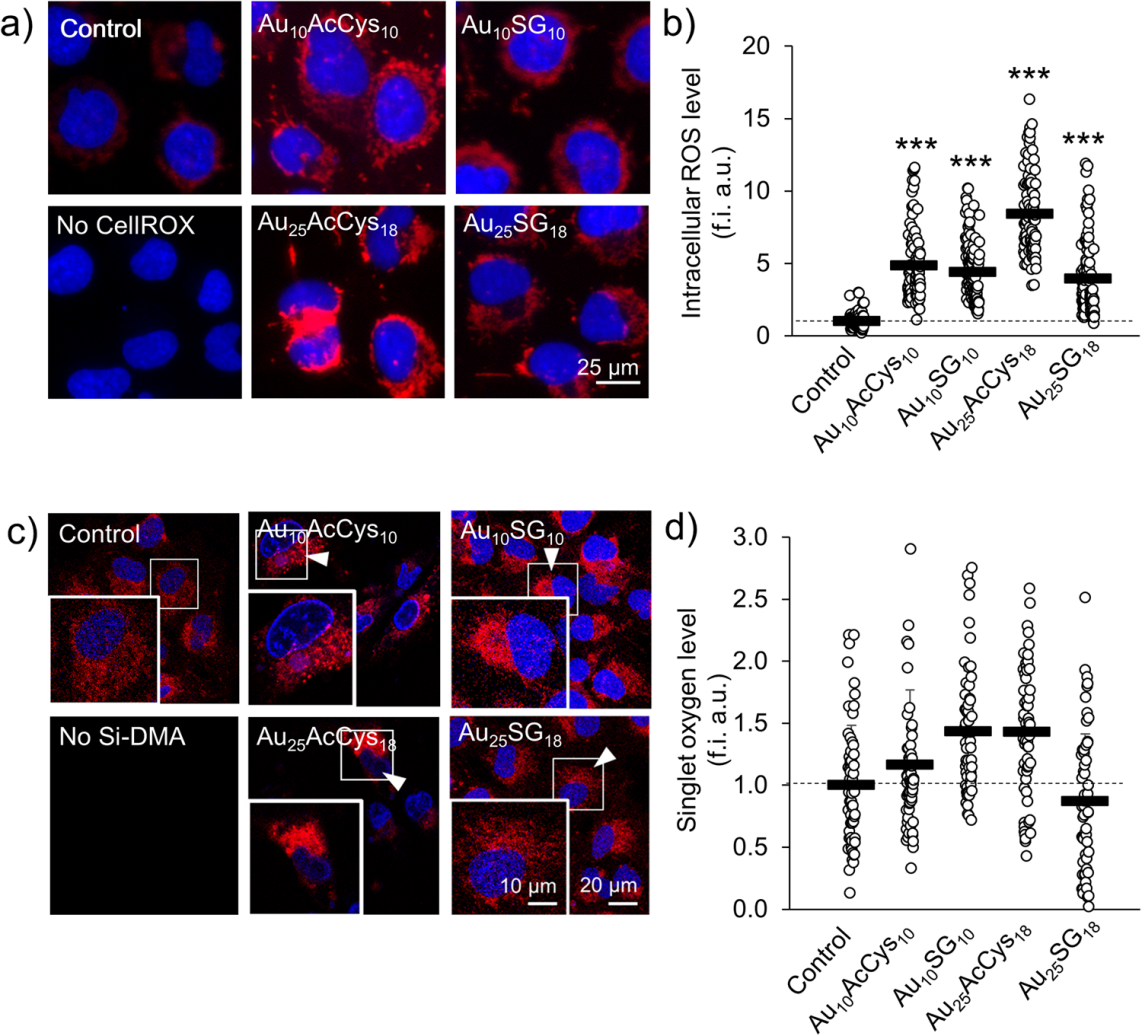
Oxidative stress in human microglia in response to single- and two-photon-stimulated gold nanoclusters. **a** Representative fluorescence micrographs of ROS level in human microglia loaded with CellROX (red) and treated with Au_10_AcCys_10_, Au_10_SG_10_, Au_25_AcCys_18_ or Au_25_SG_18_ at 100 μM in serum-deprived conditions before stimulation with a one-photon laser (473 nm) for 3 min. Nuclei (blue) were labeled with Hoechst 33342. **b** Shown are the average level of ROS per individual cell (white dot) and the average ROS level per condition (black bar ±SD) in microglia treated as in a), normalized to the fluorescence intensity of the untreated control (set to 1). f.i. a.u. fluorescence intensity arbitrary units. At least 540 cells were analyzed from three independent experiments. **c** Representative fluorescence confocal micrographs of singlet oxygen levels (red, white arrows) in human microglia treated with gold nanoclusters Au_10_AcCys_10_, Au_10_SG_10_, Au_25_AcCys_18_, or Au_25_SG_18_ at 100 μM before exposure to two-photon laser (720 nm) for 3 min to induce singlet oxygen production. Singlet oxygen level was detected using the fluorescent probe Si-DMA. Nuclei (blue) are labeled with Hoechst 33342. **d** Shown are the average level of singlet oxygen in individual microglia cells (white dot) treated as in (**a**)), and the average level per condition (black bar), normalized to the fluorescence intensity prior to laser exposure (0 min, set to 1) from at least 60 cells per condition and at least two independent experiments. ***p < 0.001.

Generation of reactive oxygen species (oxidants), including singlet oxygen, was determined in human microglia treated with the four ligated nanoclusters described above (Au_10_SG_10_, Au_10_AcCys_10_, Au_25_SG_18_, and Au_25_AcCys_18_). They did not significantly decrease cell viability after 24 h (Supplementary Fig. 6). Au_10_ generated small but detectable amounts of ROS without photoexcitation, whereas Au_25_ did not under similar conditions (Supplementary Fig. 7). In contrast, both one-photon (473 nm) and two-photon (720 nm) excitation of Au_25_AcCys_18_ causes an increase in ROS above endogenous levels. In particular, the abundance of singlet oxygen generated with two-photon excitation is markedly higher with Au_25_AcCys_18_ than with Au_25_SG_18_ (Fig. 6), complementing the theoretical part of this study.

Excessive ROS inadequately opposed by endogenous antioxidants results in oxidative stress. Several cellular mechanisms are activated in response to oxidative stress, including the master regulator of antioxidative response Nuclear factor-erythroid factor 2-related factor 2 (Nrf2). Nrf2 is a transcription factor retained in the cytosol by Kelch-like ECH-associated protein 1 (KEAP1) under basal conditions (Supplementary Fig. 8).

Oxidative stress causes Nrf2 to dissociate from KEAP1 and translocate to the nucleus to upregulate antioxidant proteins^31^. We examined the interaction of Nrf2 and KEAP1 in microglia exposed to the ligated gold nanoclusters using a proximity ligation assay, and found that Au_10_SG_10_ and Au_10_AcCys_10_, as well as Au_25_AcCys_18_, decreased the association of Nrf2 and KEAP1. The Supplementary Fig. 8b clearly shows the difference in ligand effect between SG and AcCys on Au_25_, while such ligand effect is not observed for Au_10_. SG does not contribute to the dissociation of the Nrf2-KEAP1 complex, whereas Au_25_ does. Untreated and acetyl-cysteine-treated cells served as controls. Acetyl-cysteine (100 µM or lower concentrations) did not have a significant effect on the complex dissociation (Supplementary Fig. 8b).

## Discussion

In this study, we show that both the molecular-like properties of ultrasmall nanoclusters, as well as the nature of ligands affect the efficiency of gold clusters in solution to produce singlet oxygen upon excitation with visible light in a one-photon regime. Au_25_ nanoclusters with a gold core (and thus confined electrons) have a higher efficiency in generating ^1^O_2_ than Au_10_ catenane structures with zero confined electrons. This behavior may be explained by (i) the excitation wavelength better matches the position of first singlet states in Au_25_ than in Au_10_. In other words, the optical energy gap is inversely proportional to the number of confined electrons (the larger the number of confined electrons in the gold core, the smaller the optical gap)^32^. (ii) the S-T gap in Au_25_ is lower than in Au_10_, making the intersystem crossing (ISC) process easier for Au_25_. (iii) the energy gap (T_1_-S_0_) better matches the one of ligated Au_25_ than that of Au_10_ with the 0.98 eV energy required to generate ^1^O_2_, thus facilitating the energy transfer process for Au_25_.

The ligand nature also plays an important role in the capability to produce ^1^O_2_ upon photoexcitation of nanoclusters. Acetyl-cysteine is more efficient than glutathione in attenuating ^1^O_2_. This difference may be due to the structural motifs of surface ligands, in particular H-bond formation. Since glutathione is bulky and possesses both carboxylic groups and amine groups, a rich H-bond network formation is facilitated (Figs. 2 and 3), allowing better protection from solvent exposure^33^. The effect of a larger number of H-bonds is directly related to the flexibility of the ligated nanoclusters. Gold nanoclusters with acetyl-cysteine ligands are more flexible due to the smaller number of H-bonds, which enhances flexibility, thus increasing the probability of ISC between S and T states. This greater flexibility will induce a more efficient non-radiative relaxation and thus allowing for more efficient ISC between S and T states. Results from single-cell analyses upon treatment with the gold nanoclusters show that (1) Au_10_ is able to produce ROS with and without photoexcitation, but Au_25_ is able to produce ROS only with photoexcitation. (2) Formation of ROS leads to changes in the protein association between Nrf2 and KEAP1. Nrf2 is translocated to the nucleus when it dissociates from KEAP1^34,35^. This translocation turns on the activation of antioxidant genes as a protective mechanism against oxidative stress. We selected SG and acetyl-cysteine as ligands because these are endogenous compounds with established antioxidant effects^36,37^. These small compounds do not disturb cellular functions, as they are present endogenously in high concentrations. Interestingly, when attached to the gold nanoclusters, particularly Au_25_, they show different effects. Possible interpretations for differences in biological effects in the production of ROS and Nrf2-KEAP1 response could be ascribed to the greater flexibility of Au_25_AcCys_18_ compared to Au_25_SG_18_. In line with the computational findings, surface ligands provide considerably greater amount of H-bonds in the case of Au_25_SG_18_ than with Au_25_AcCys_18_. This could result in the detachment of AcCys from the Au_25_ gold core more easily than for the surface ligand of Au_25_SG_18_. It is well documented that weakly attached ligands are replaced by intracellular SG, which is present in the 1–5 mM range.

We would like to point out here that experiments in solution and in cells were done with one- and two-photon excitation regimes, respectively. With two-photon excitation (at 720 nm, thus 1.72 eV), the terminal energy (e.g. 3.44 eV) would permit higher absorption for Au_10_ nanoclusters, thus opening more efficiently channels for de-excitation and the ISC process (compared to visible single-photon excitation). We cannot exclude that the relaxation pathways following one-photon excitation are different than that of two-photon excitation, as we demonstrated for the luminescence properties of Ag_29_ nanoclusters^38^. Such higher absorption and thus the opening of other channels for de-excitation and the ISC process might explain the singular behaviors observed in cells for Au_10_ nanoclusters— in particular Au_10_SG_10_. Clearly, a quantitative comparison of ^1^O_2_ generation rate by Au_10_ and Au_25_ with two-photon excitation in solution would merit to be conducted. We managed to adapt the indirect method to measure ROS of nanoclusters in solution in the NLO regime. For this purpose, we verified the photostability of DPBF under high power femtosecond irradiation (see Supplementary Fig. 9). Then, we conducted ROS measurements in solution using the indirect method laser irradiation at 780 nm (for Au_25_SG_18_, Au_25_AcCys_18_ and Au_10_SG_10_) and at 720 nm (for Au_25_SG_18_, Au_25_AcCys_18_). To our knowledge, this is the first measurements of ROS efficiency in the NLO regime for nanoclusters. The ^1^O_2_ generation rates of gold nanoclusters under a pulse laser irradiation at 780 nm and 720 nm are presented in Supplementary Table 2 (and Supplementary Fig. 10). Amazingly, both Au_10_ and Au_25_ NCs present efficient singlet oxygen generation rate under pulse 780 nm and 720 nm irradiation, and trends upon two-photon excitation (780 nm and 720) are similar to the one observed upon one-photon excitation (473 nm). In sum, our study presents an unprecedented, in-depth analysis of events taking place within gold nanoclusters when photoexcited from solution, to their possible biological consequences in living cells.

The principal aim of this study was to evaluate the possible ligand impact on ROS generation of photoexcited Au_10_ and Au_25_ nanoclusters. However, unopposed excess ROS leads to oxidative stress and deleterious effects in cells, which can be detected not only at/in the cell membranes, but also as diminished mitochondrial metabolic activity associated with morphological abnormalities, protein misfolding and aggregation, nuclear chromatin condensation, and shrinkage or disruption of the nuclear integrity. The choice of ligands (SG or AcCys) as protecting agents for gold clusters was mainly driven by their biocompatibility and their similarity from a biological point of view. In addition, our experimental-theoretical investigation has allowed to gain better insight into key factors (energetics of excited states (see Supplementary Table 3) and structural influence of surface ligands, in particular through hydrogen bonding networks) and their relative importance involved in singlet oxygen formation upon photoexcitation in the visible range. Altogether, this joint theoretical and experimental study allows to propose Au_25_AcCys_18_ ligated nanocluster as a good candidate for PDT. We also carried out live cell imaging to explore the ability of Au_10_ and Au_25_ nanoclusters to generate ROS in cells by one- and two-photon excitation. A deeper insight into the impact of nanoclusters on cell components (e.g. protein association between Nrf2 and KEAP1) was also reported.

A triangular strategy consisting of ligated photoexcited gold nanoclusters in solution and in living cells, combined with a theoretical approach for the structural and photophysical properties of nanoclusters with explicit ligands has been presented. This allowed for in-depth exploration of ROS generation by photoexcited nanoclusters at atomic precision, opening new routes for applications in PDT. Our studies focused on singlet oxygen species in living microglia cells, which surround the neurons and constitute a microenvironment of brain tumors. PDT employing Au_25_ nanoclusters would be of particular interest for brain tumor ablation without strongly disturbing the homeostasis of the microenvironment. To prove the usefulness of Au_25_AcCys_18_ for such a purpose, organoids with human cells would be an attractive biological model.

## Methods

### Au_10_ and Au_25_ cluster synthesis

All chemicals were commercially available and used without purification. HAuCl_4_·3H_2_O, N-acetyl-L-cysteine (AcCys), and 1,3-diphenylisobenzofuran (DPBF) were purchased from Sigma-Aldrich. L-glutathione reduced, tributylamine, and triethylamine were procured from Carl Roth. Sodium borohydride was purchased from ACROS ORGANICS. Ammonia (NH_4_OH), diethyl ether (Et_2_O), and ethanol were purchased from VWR Chemicals. Methanol (MeOH) was purchased from Honeywell. New methylene blue (NMB) was purchased from TCI. Ultrapure Milli-Q water (resistivity 18.2 MΩ) was used for experimental purposes.

Au_10_SG_10_ and Au_25_SG_18_ nanoclusters were synthesized as reported by Bertorelle et al.^19^ and by Ji et al.^39^, respectively. Au_10_AcCys_10_ was synthesized as follow. 125 mg of L-Glutathione was dissolved in 35 mL of methanol and 2 mL of triethylamine. 100 mg of HAuCl_4_·3H_2_O in 15 mL of water was added and the solution was stirred overnight at ambient temperature. To complete precipitation, MeOH/Et_2_O (volume rate 1:1) was added till precipitation. The dispersion was centrifuged. The powder was dissolved in a minimum of H_2_O/NH_4_OH solution and then precipitated with MeOH/Et_2_O. The unwanted products were removed with cycles of dissolution/precipitation/centrifugation. After centrifugation, the powder was dissolved again in 10 mL of water. Then, 2 mL of glacial acetic acid was added and the solution was left undisturbed for 1 h. Pure Au_10_AcCys_10_ was precipitated and collected by centrifugation. A last cycle of dissolution/precipitation/centrifugation with H_2_O/NH_4_OH – MeOH/Et_2_O was done before drying the powder.

For Au_25_AcCys_18_ synthesis,100 mg of gold salts (HAuCl_4_·3H_2_O) was added to a solution of acetyl-cysteine (234 mg) dissolved in methanol (35 mL), followed by adding tributylamine (2 mL) and triethylamine (2 mL). After stirring for 5 min at room temperature, a first reducing agent was added (sodium borohydride, 3 × 25 mg spaced by 30 min). Then water (15 mL) and diethyl ether (15 mL) were added, followed by the addition of a second reducing agent (sodium borohydride, 4 × 50 mg spaced by 30 min). The solution was left undisturbed overnight before purification. Precipitation was induced by adding NH_4_OH (1 mL, 10%), and the solution was centrifuged (9000 rpm). The unwanted products were removed with cycles of dissolution/precipitation/centrifugation. The powder was dissolved in a minimum of H_2_O/NH_4_OH, then precipitated with MeOH. After centrifugation, the powder was dissolved in water (10 mL), followed by adding glacial acetic acid (2 mL), then the solution was left undisturbed for 1 h before being centrifuged. The supernatant was collected and precipitated with MeOH. A last cycle of dissolution/precipitation with H_2_O/NH_4_OH and MeOH was done before drying the powder under vacuum.

### Singlet oxygen generation and detection in solution

A 473 nm continuous wavelength laser (Changchun New Industries Optoelectronics Tech. Co., Ltd, China) with an output power of 250 mW and a beam diameter of 3 mm was used to photoexcite the nanoclusters in the linear optical regime. In the NLO regime, a Ti:Sapphire femtosecond laser (Coherent, Chameleon Ultra I) operating at 780 nm (720 nm) was used for two photon excitation with an irradiation power of 2.26 W (1.13 W) and a beam diameter of 1.2 mm. A typical solution used in the experiments contained nanoclusters and DPBF with a concentration of 1.37 × 10^−6^ M and 6.15 × 10^−5^ M, respectively. All solutions were prepared in ethanol. The samples were loaded in quartz cuvettes (1 cm light path length). Absorption spectra were recorded after photoexcitation of the nanoclusters with different times. The concentration of DPBF was calculated from the intensity of absorption peak at 412 nm according to the Beer–Lambert law. UV−vis absorption spectra were recorded on an Avantes AvaSpec-2048 spectrophotometer with an AvaLight DH-S deuterium lamp. Fluorescence emission spectra were recorded with a Horiba FluoroMax-4 spectrophotometer. Fluorescence lifetime was measured on a custom-built set-up^40^. The quantum yields of Au_10_AcCys_10_ and Au_25_AcCys_18_ were measured using Au_10_SG_10_ and Au_25_SG_18_ as references, respectively.

### Cell culture

HMC3 human microglia were originally received from the American Type Culture Collection (ATCC). Unless otherwise indicated, cells were cultured in Dulbecco’s Modified Eagle Medium (DMEM, Thermo Fisher Scientific) with 5% (v/v) fetal bovine serum (Wisent) and 1% (v/v) penicillin-streptomycin. Cells are kept at 37 °C with 5% CO_2_ and 95% relative humidity. Cells tested negative for mycoplasma contamination.

### Reactive oxygen species with one-photon stimulation

Reactive oxygen species in microglia stimulated with a one-photon laser was measured in cells seeded onto 12 mm glass coverslips (Assistant) at 7000 cells per coverslip, and cultured for 24 h. Cells were loaded with CellROX Deep Red (5 μM, Thermo Fisher Scientific) and Hoechst 33342 (10 μM, Millipore-Sigma) for 30 min at 37 °C, in phenol- and serum-free DMEM (Thermo Fisher Scientific). Cells were washed once in phenol- and serum-free DMEM before treatment with gold nanoclusters, and then exposure to a mercury laser (473 nm) for 3 min. Cells were imaged 5 min following laser exposure with a fluorescence microscope (Leica DMI4000 B). Fluorescence was analyzed in ImageJ (version 1.53t).

### Singlet oxygen with two-photon stimulation

Microglia were seeded into 60 mm culture dishes (Fisher Scientific) at 20,000 cells per dish, and cultured for 24 h. Cells were washed twice with phosphate-buffered saline (PBS) before incubation with Si-DMA (50 nM, Thermo Fisher Scientific)^41,42^ and Hoechst 33342 (10 μM) for 30 min at 37 °C in phenol-free Hank’s Balanced Salt Solution (Thermo Fisher Scientific). Cells were washed once with Hank’s Balanced Salt Solution, then treated with gold nanoclusters before stimulation with a Coherent Chameleon titanium-sapphire Multiphoton V2 laser IR two-photon laser (720 nm, 10% intensity, 80 MHz) for 3 min. Images were taken 5 min after laser exposure, with an argon 638 nm laser of a confocal microscope (Leica SP8). Fluorescence was analyzed in ImageJ.

### Reactive oxygen species without photostimulation

Microglia were seeded onto 12 mm glass coverslips (Assistent) at 7000 cells per coverslip, and cultured for 24 h. Cells were washed twice with PBS before treatment with gold nanoclusters in serum-free DMEM. After treatment, cells were incubated with CellROX Deep Red (5 μM, Thermo Fisher Scientific) and Hoechst 33342 (10 μM, Millipore-Sigma) for 30 min at 37 °C, in phenol- and serum-free DMEM (Thermo Fisher Scientific). Cells were washed once in phenol- and serum-free DMEM before imaging with a fluorescence microscope (Leica DMI4000 B). Fluorescence was analyzed in ImageJ (version 1.53t).

### Proximity ligation assay

Microglia were seeded and treated as for the detection of reactive oxygen species. After treatment, cells were washed twice with PBS and fixed with 4% (w/v) paraformaldehyde (BDH) for 10 min. Cells were permeabilized with 0.1% (v/v) Triton X-100 (Millipore-Sigma) in PBS for 10 min. The proximity ligation assay was performed following the manufacturer’s protocol for Duolink (Millipore-Sigma) and using primary antibodies against Nrf2 (rabbit, 1/500, ab31163, Abcam) and KEAP1 (mouse, 1/500, 4G10H9, Proteintech) for 24 h at 4 °C. Cells were then incubated with Phalloidin Alexa Fluor 488 (1X, Thermo Fisher Scientific) and Hoechst 33342 (10 μM) for 20 min in PBS, washed twice with PBS, and mounted onto microscope slides (Fisher Scientific) using Aqua-Poly/Mount (Polysciences). Cells were imaged using a fluorescence microscope (Leica DMI4000 B).

### Cell viability

Cells were seeded onto glass coverslips at 10,000 cells per coverslip and incubated for 24 h before treatment. Cells were treated with gold nanoclusters as for the measurement of reactive oxygen species. After treatment, cells were fixed with 4% paraformaldehyde (10 min), permeabilized with 0.1% Triton X-100 (10 min), and nuclei were labeled with Hoechst 33342 (10 μM, 10 min). Cells were washed twice with PBS, then mounted onto microscope slides using Aqua-Poly/Mount. Cells were imaged using a fluorescence microscope (Leica DMI4000 B).

### Statistics

One-way ANOVA with Tukey-Kramer’s post-hoc test was performed. In accordance to the Central Limit Theorem, sample sizes larger than 30 were assumed to have a normal distribution. Equality of variance was verified by Levene’s test. A *p*-value lower than 0.05 indicated statistical significance.

### Computational approach

Density functional theory (DFT) has been used to determine the structural properties of liganded AuNCs: Au_25_(SCH3)_18_ and Au_10_(SCH_3_)_10_. The optimization of structures was performed with PBE functional^43,44^ implemented in Gaussian computational chemistry software. Coordinates for the starting structure of Au_25_(SCH3)_18_ were taken from crystal structure of Au_25_(SCH2CH2Ph)_18_^45^ as well as from previously optimized DFT structure^46^, and the coordinates for the starting structure of Au_10_(SCH_3_)_10_ were taken from previously optimized DFT structure^19,47^. Semiempirical method PM7^48^ has been used to optimize the AuNCs structures with full ligands, by freezing the coordinates of gold and sulfur atoms to ensure that Au-Au and Au-S bond distances remain unchanged with respect to previously obtained bond distances from DFT structures of Au_10_(SCH_3_)_10_ and Au_25_(SCH3)18. Structures with full ligands were used in calculations of excited state properties within time-dependent density functional (TDDFT) method. Relativistic effective core potential^46^ of the Stuttgart group was employed for gold atoms. For gold and sulfur atoms SVP AO basis set^49^, and for other atoms of ligands 3-21 G AO’s basis set was used^50,51^. Singlet and triplet states were obtained using the TDDFT and Coulomb-attenuated version of Becke’s three-parameter non-local exchange functional together with the Lee–Yang–Parr gradient-corrected correlation functional^52^ implemented in Gaussian^53^. Restricted TDDFT approach has also been employed for calculation of triplet states. The ΔE_S-T_ obtained from unrestricted TDDFT show the same trend: ΔE_S-T_ is larger for Au_10_SG_10_, Au_10_AcCys_10_, then for Au_25_SG_18_, Au_25_AcCys_18_. Size effect due to structural difference is dominant. Effect of different ligands on the given cluster size is negligible for linear optical properties because ligands do not participate in excitations. Consideration of organization of ligands using molecular dynamic simulations is relevant in the context of aggregation and fibrillation^54,55^. Inclusion of solvent doesn’t have major impact on geometries since the changes in average distance are not larger than 0.010 Å.

## Supplementary information

Supplemental Information

## Data Availability

The data supporting the findings of this study are available upon reasonable request from the corresponding authors.
