# Supplementary material for: Ligand impact on reactive oxygen species generation of Au10 and Au25 nanoclusters upon one- and two-photon excitation
Source: Commun Chem. 2023 May 22;6:97. doi: 10.1038/s42004-023-00895-5 (PMC10203263; doi:10.1038/s42004-023-00895-5)
Supplement: Supplementary file 1 — Supplemental Information [file 42004_2023_895_MOESM1_ESM.pdf]

Supplementary Information

Ligand impact on reactive oxygen species generation of Au_{10} and Au_{25} nanoclusters upon one- and two-photon excitation

Hussein Fakhouri[1,2], Martina Perić Bakulić[2,3], Issan Zhang[4], Hao Yuan[1], Dipankar Bain[1], Fabien Rondepierre[1], Pierre-François Brevet[1], Željka Sanader Maršić[5], Rodolphe Antoine[1,*], Vlasta Bonačić-Koutecký[2,6,7,*] and Dusica Maysinger[4,*]

[1]Institut Lumière Matière, CNRS UMR 5306, Université Claude Bernard Lyon 1, Univ. Lyon, 69622 Villeurbanne Cedex, France

[2]Center of Excellence for Science and Technology, Integration of Mediterranean Region (STIM), Faculty of Science, University of Split, Ruđera Boškovića 33, 21000 Split, Croatia

[3]Faculty of Chemistry and Technology, University of Split, Rudera Boskovica 35, 21000 Split, Croatia

[4]Department of Pharmacology and Therapeutics, McGill University, 3655 Promenade Sir-William-Osler, H3G 1Y6 Montreal, Canada

[5]Faculty of Science, University of Split, Rudera Boskovica 33, 21000 Split, Croatia

[6]Interdisciplinary Center for Advanced Science and Technology (ICAST), University of Split, Meštrovićevo šetalište 45, 21000 Split, Croatia

[7]Chemistry Department, Humboldt University of Berlin, Brook-Taylor-Strasse 2, 12489 Berlin, Germany

* These authors jointly supervised this work.

Correspondence

rodolphe.antoine@univ-lyon1.fr , vbk@cms.hu-berlin.de and

dusica.maysinger@mcgill.ca

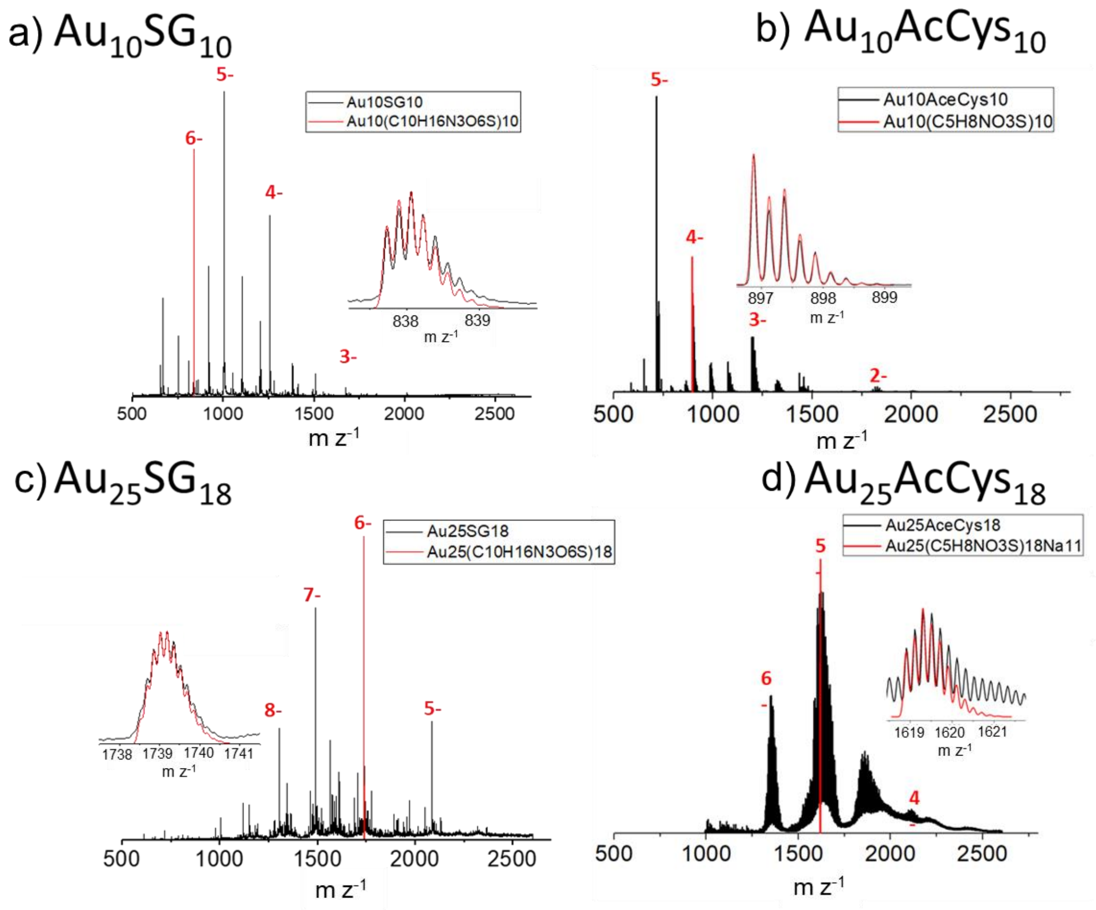

Supplementary Figure 1. Mass spectra of Au_{10} and Au_{25} with N-acetyl-cysteine (AcCys) and glutathione (SG) ligands. ESI-MS spectra of **a)** $Au_{10}SG_{10}$, **b)** $Au_{10}AcCys_{10}$, **c)** $Au_{25}SG_{18}$, and **d)** $Au_{25}AcCys_{18}$ with simulation of isotope pattern. Major peaks correspond to different charge states of nanoclusters. Sodium adducts are present in the mass spectra of $Au_{25}AcCys_{18}$.

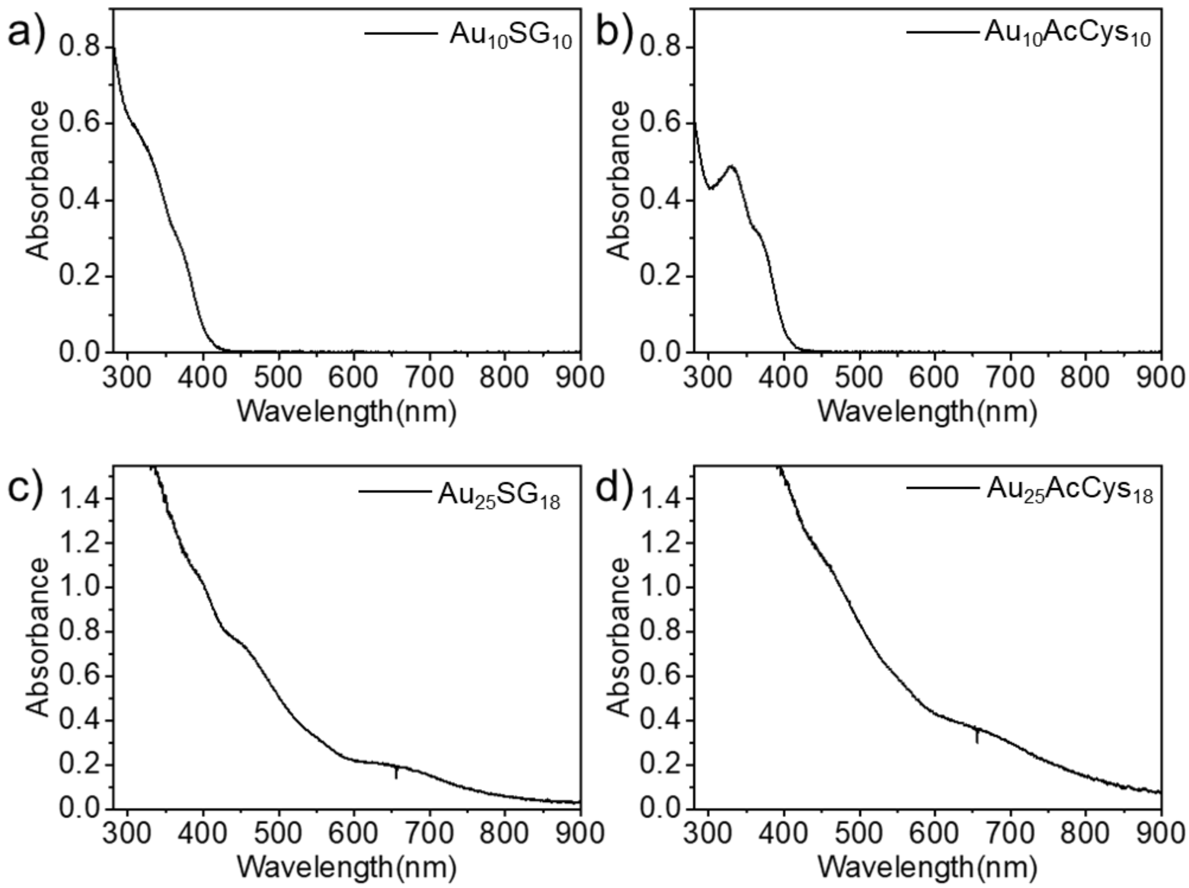

Supplementary Figure 2. Absorption spectra of gold nanoclusters. UV-vis spectra of **a)** $Au_{10}SG_{10}$, **b)** $Au_{10}AcCys_{10}$, **c)** $Au_{25}SG_{18}$, and **d)** $Au_{25}AcCys_{18}$ at the concentration of 4×10^{-5} M.

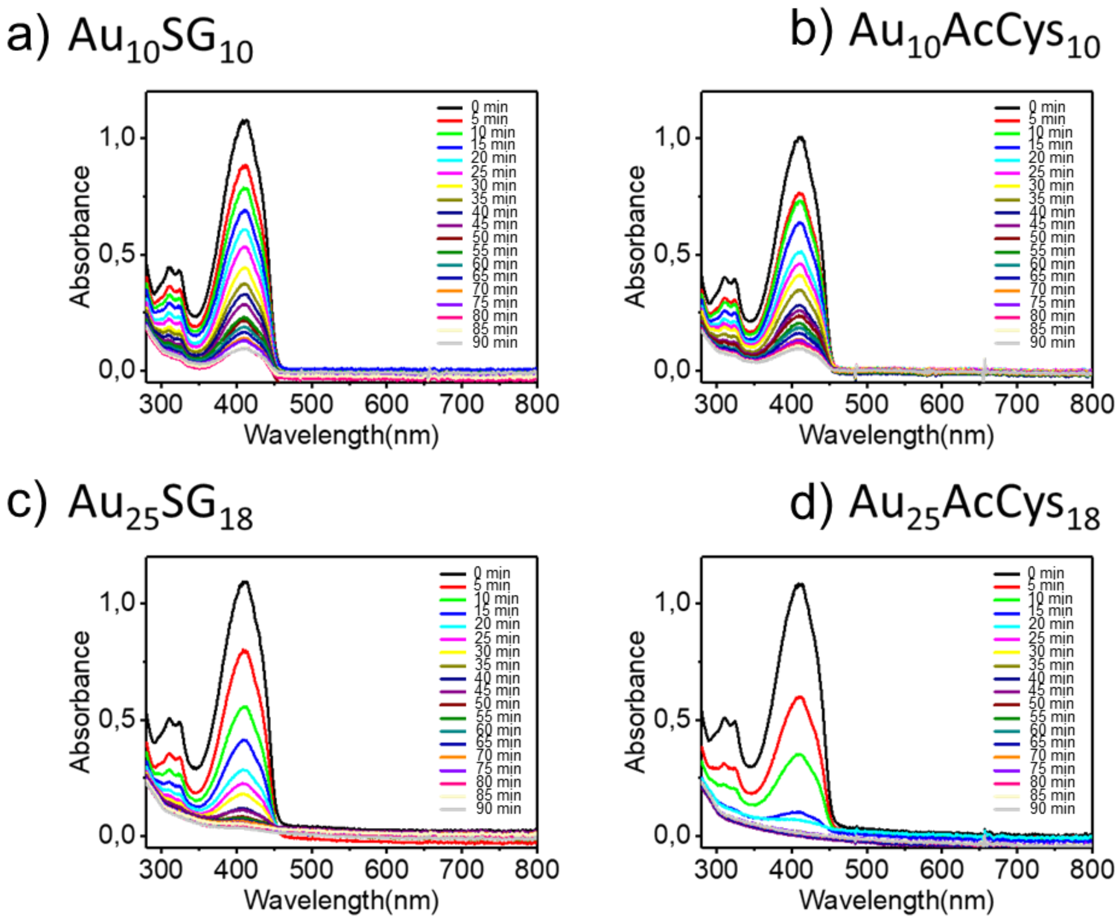

Supplementary Figure 3. One-photon-excited singlet oxygen generation in solution of gold nanoclusters detected by 1,3-diphenylisobenzofuran (DPBF). Evolution of DPBF absorbance spectra in function of time under continuous wave laser (473 nm) irradiation with **a)** $Au_{10}SG_{10}$, **b)** $Au_{10}AcCys_{10}$, **c)** $Au_{25}SG_{18}$, and **d)** $Au_{25}AcCys_{18}$.

Supplementary Table 1. Normalized $^1O_2$ generation rate of gold nanoclusters and new methylene blue (NMB) by absorbance at 473 nm.

Photosensitizer	Absorption at 473 nm (4 x 10^{-5} M)	$^1O_2$ generation rate ($^1O_2$ per cluster per min) @473 nm	Normalized rate @473 nm
Au10SG10	0.003	1.070±0.067	357
Au10AcCys10	0.003	1.094±0.183	365
Au25SG18	0.64	1.857±0.057	2.9
Au25AcCys18	1.01	2.708±0.198	2.7
NMB	0.073	2.276±0.106	31.1

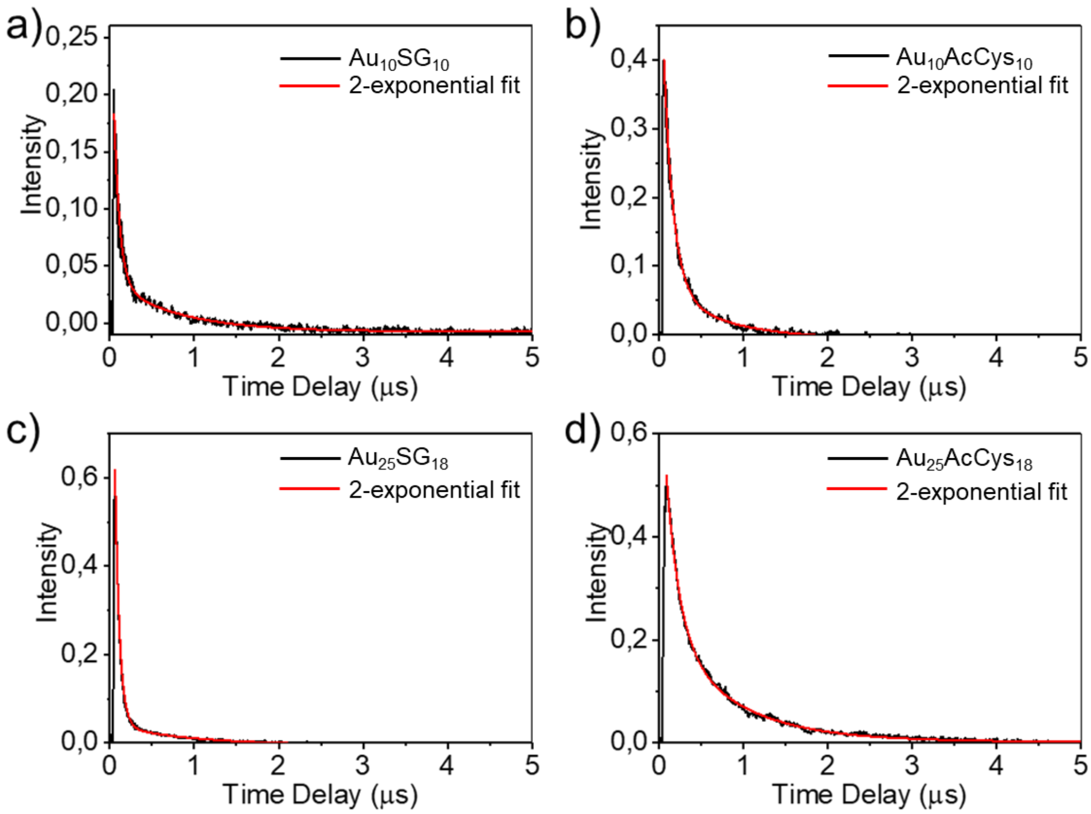

Supplementary Figure 4. Lifetime of gold nanoclusters. Photoluminescence decay curves of **a)** $Au_{10}SG_{10}$, **b)** $Au_{10}AcCys_{10}$, **c)** $Au_{25}AcCys_{18}$, and **d)** $Au_{25}SG_{18}$. Red lines are curves fitted by two-exponential decay function.

a) $Au_{10}AcCys_{10}$

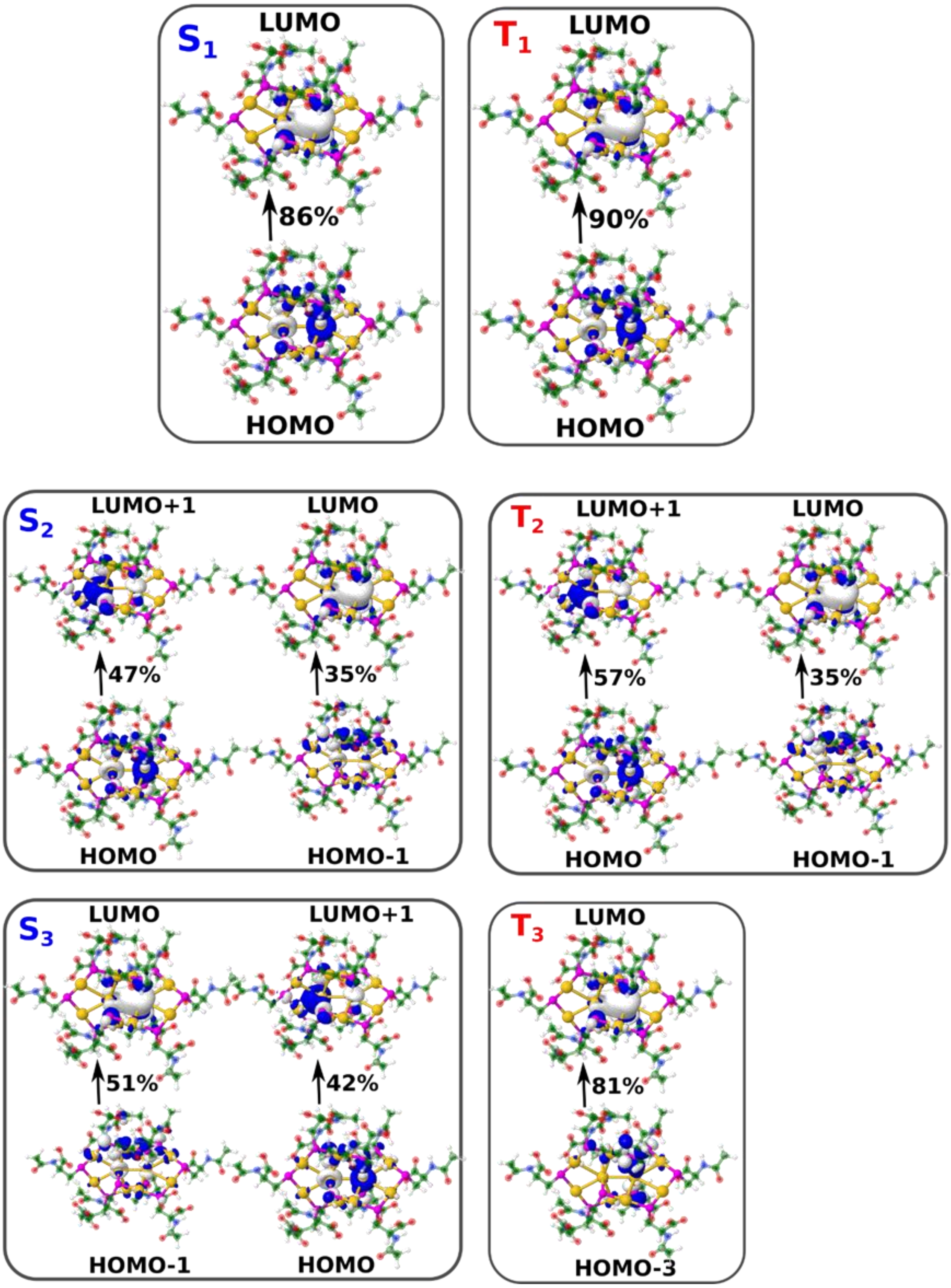

b) $Au_{10}SG_{10}$

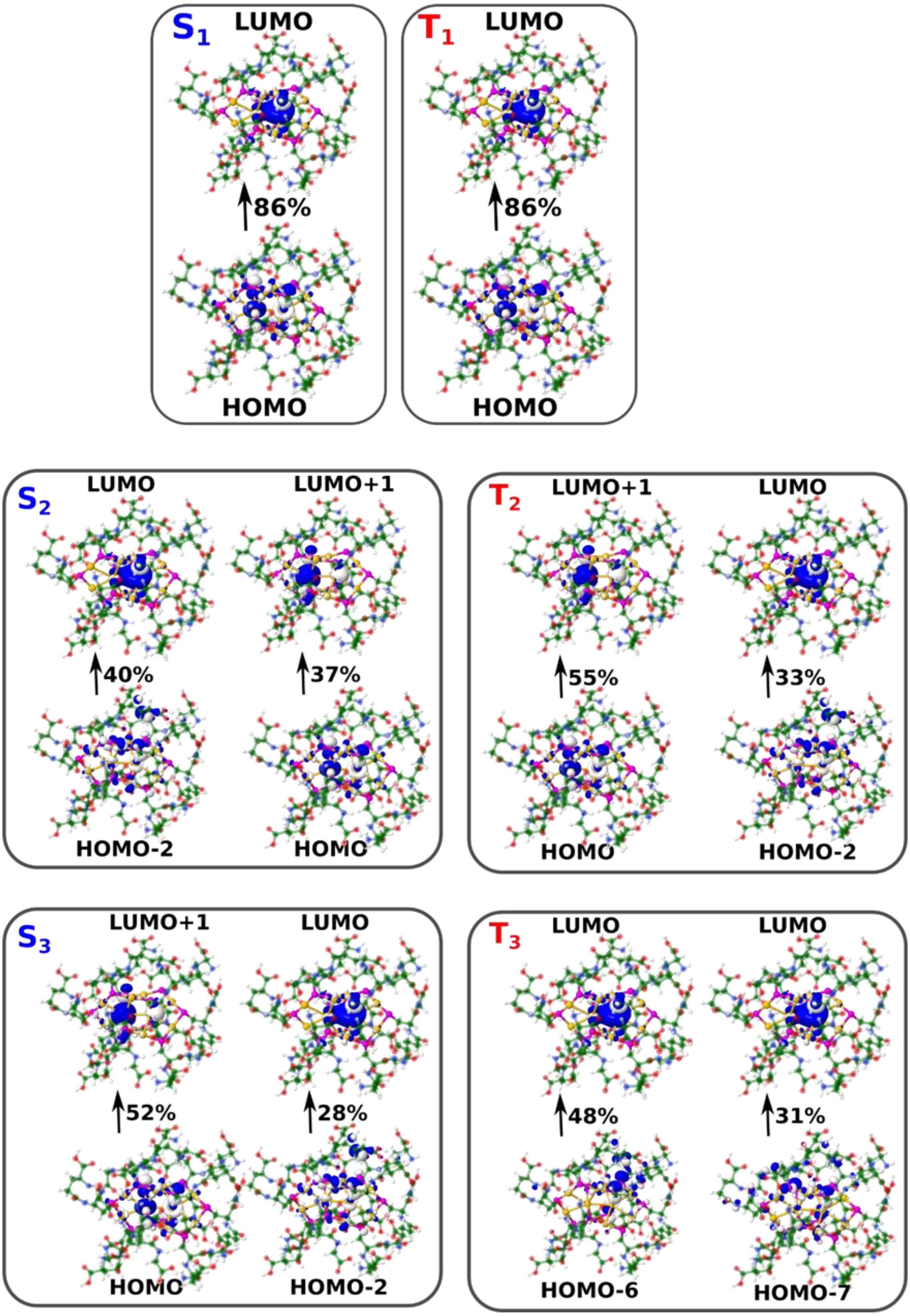

S1 LUMO
86%
HOMO
T1 LUMO
86%
HOMO
S2 LUMO LUMO+1
40% 37%
HOMO-2 HOMO
T2 LUMO+1 LUMO
55% 33%
HOMO HOMO-2
S3 LUMO+1 LUMO
52% 28%
HOMO HOMO-2
T3 LUMO LUMO
48% 31%
HOMO-6 HOMO-7

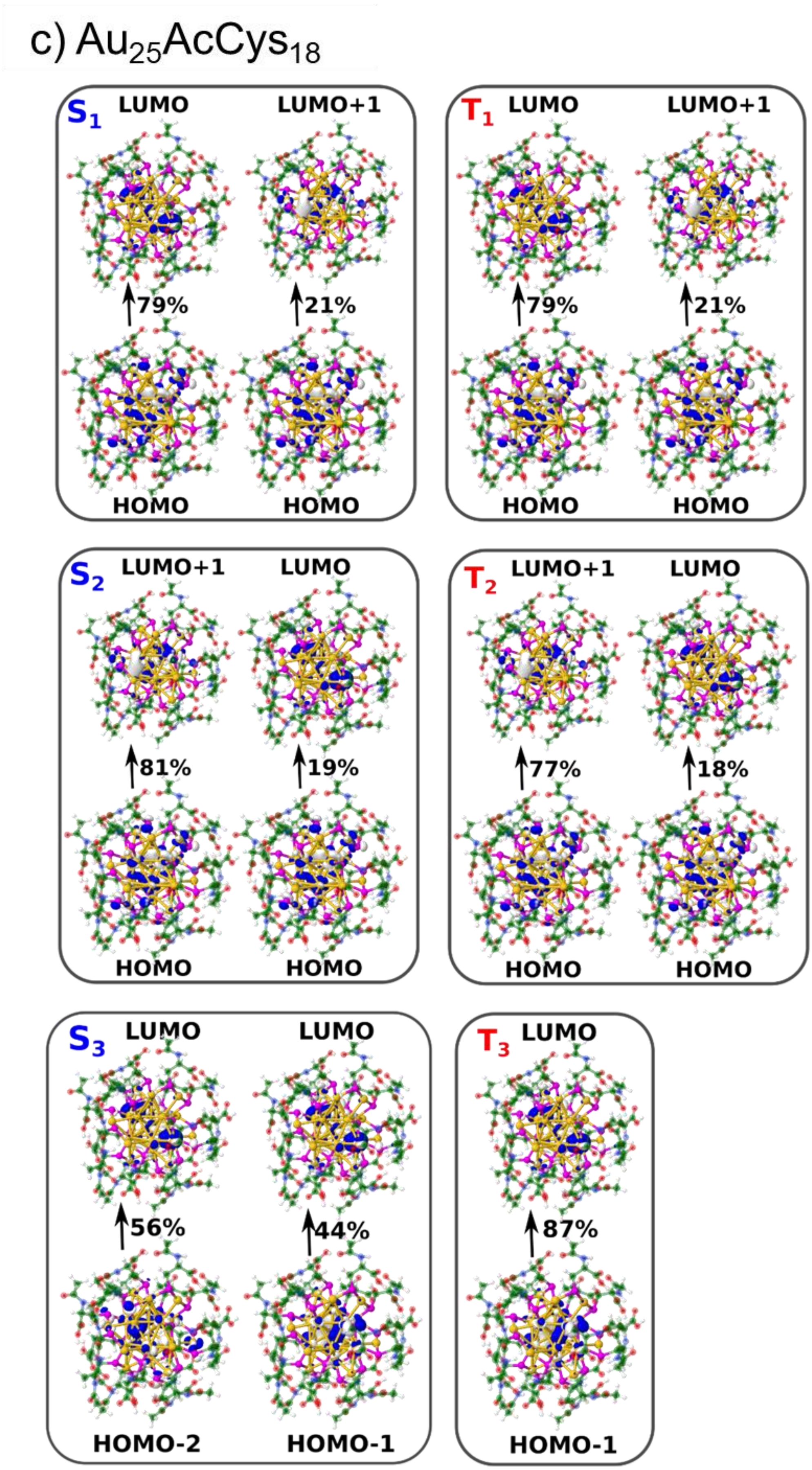
c) $Au_{25}AcCys_{18}$
S1
LUMO
LUMO+1
79%
21%
HOMO
HOMO
T1
LUMO
LUMO+1
79%
21%
HOMO
HOMO
S2
LUMO+1
LUMO
81%
19%
HOMO
HOMO
T2
LUMO+1
LUMO
77%
18%
HOMO
HOMO
S3
LUMO
LUMO
56%
44%
HOMO-2
HOMO-1
T3
LUMO
87%
HOMO-1

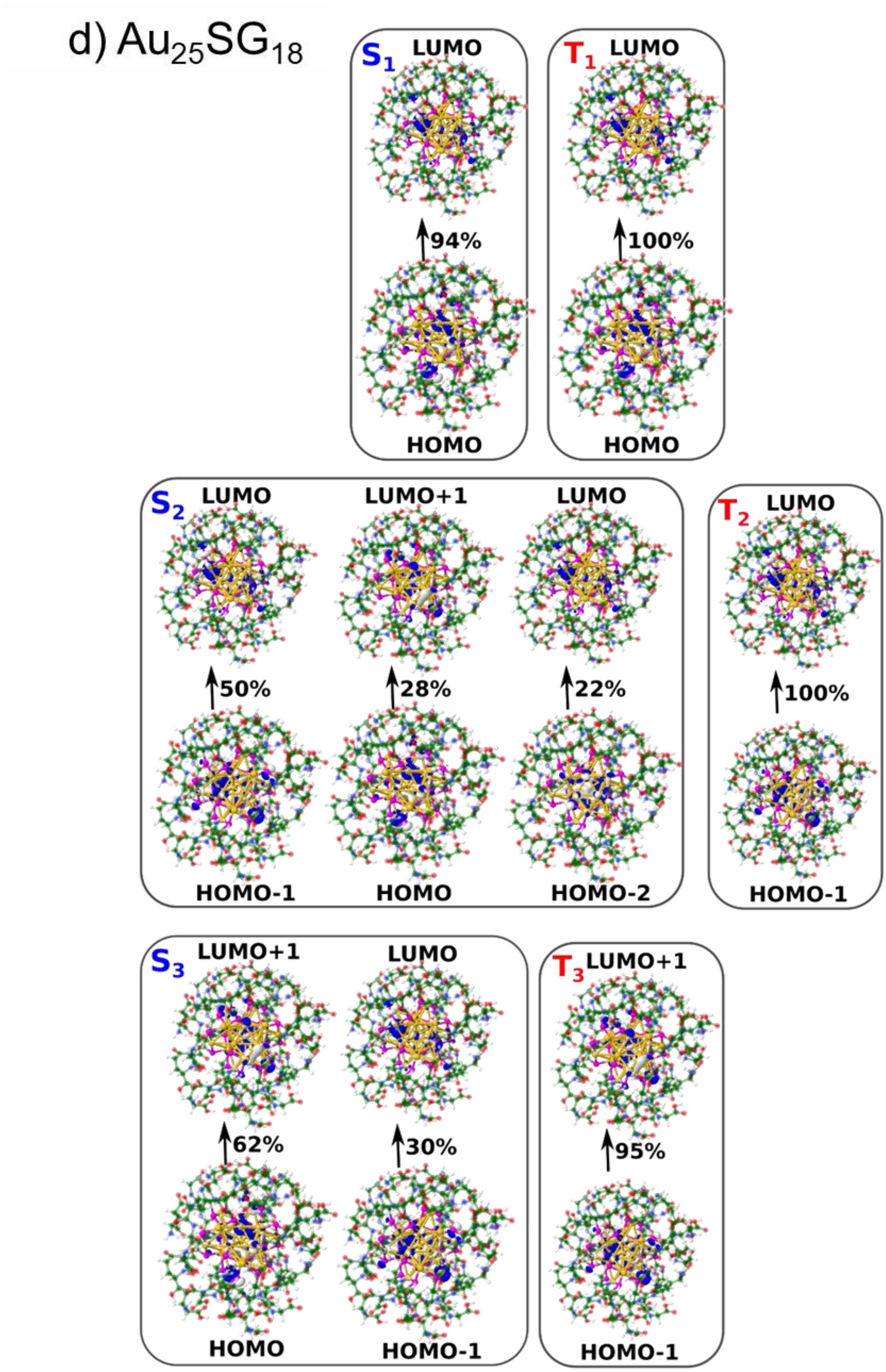

Supplementary Figure 5. Analysis of leading excitations among molecular orbitals within lowest lying singlet and triplet states. Analyses are shown for **a)** $Au_{10}AcCys_{10}$, **b)** $Au_{10}SG_{10}$, **c)** $Au_{25}AcCys_{18}$, and **d)** $Au_{25}SG_{18}$. Gold atoms are labeled yellow and ligands are shown in Figure 2.

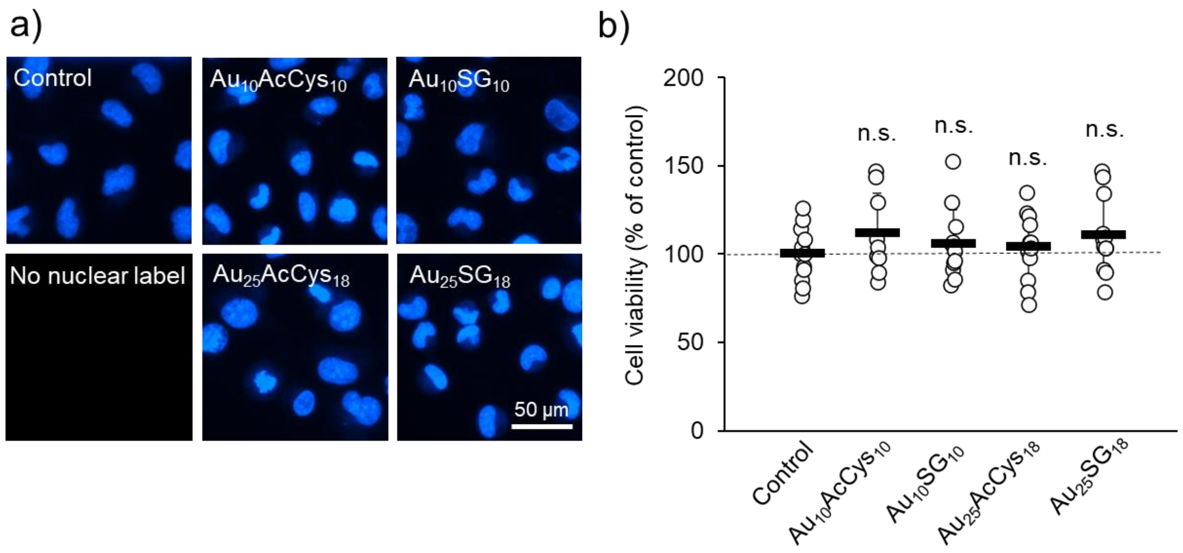

Supplementary Figure 6. Gold nanoclusters do not decrease human microglia viability after 24h. a) Representative fluorescence micrographs of cell nuclei from human microglia treated with gold nanoclusters $Au_{10}AcCys_{10}$, $Au_{10}SG_{10}$, $Au_{25}AcCys_{18}$ or $Au_{25}SG_{18}$ at 10 µM for 24h in serum-deprived conditions. Nuclei (blue) were labeled with Hoechst 33342. Cells were imaged using a fluorescence microscope. **b)** Shown are the average percentage (black bar) ±SD cell viability of human microglia treated as in **a)**, normalized to the untreated control (set to 100%). At least 350 cells were analyzed per experiment, from at least three independent experiments. n.s. non-significant.

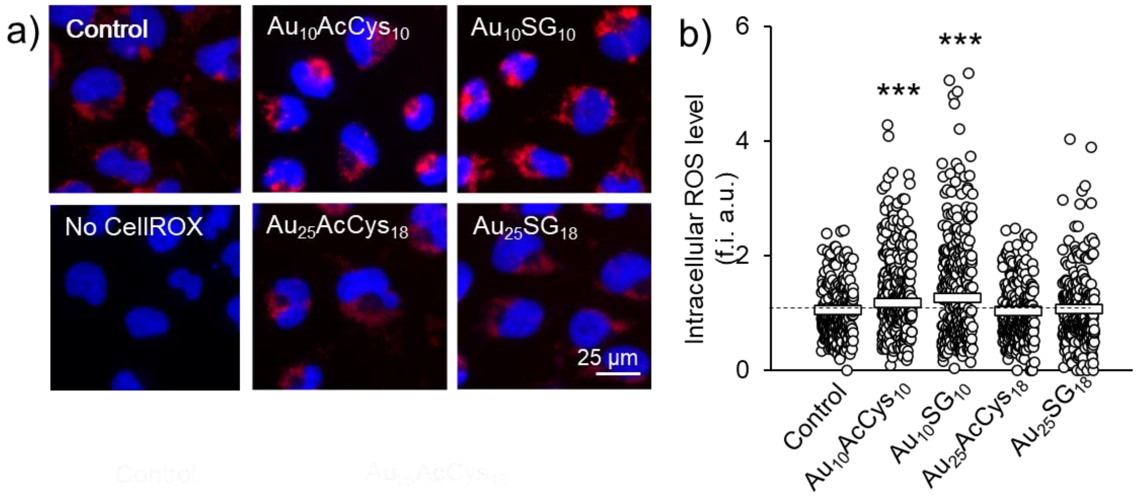

Supplementary Figure 7. Oxidative stress in human microglia without photoexcitation. a) Representative fluorescence micrographs of reactive oxygen species (ROS) level in human microglia treated with gold nanoclusters. Cells were treated with $Au_{10}AcCys_{10}$, $Au_{10}SG_{10}$, $Au_{25}AcCys_{18}$ or $Au_{25}SG_{18}$ at 10 µM for 24h in serum-deprived conditions. ROS (red) was detected using the fluorescent probe CellROX. Nuclei (blue) are labelled with Hoechst 33342. Unlabelled cells served as negative control. **b)** ROS level in human microglia treated as in **a)**. Shown are the ROS level per individual cell (white dot) and the average ROS level per condition (white bar ±SD), normalized to the fluorescence intensity of the untreated control (set to 1). f.i. a.u. fluorescence intensity arbitrary units. At least 300 cells were analyzed per condition from at least three independent experiments. ***$p<0.001$.

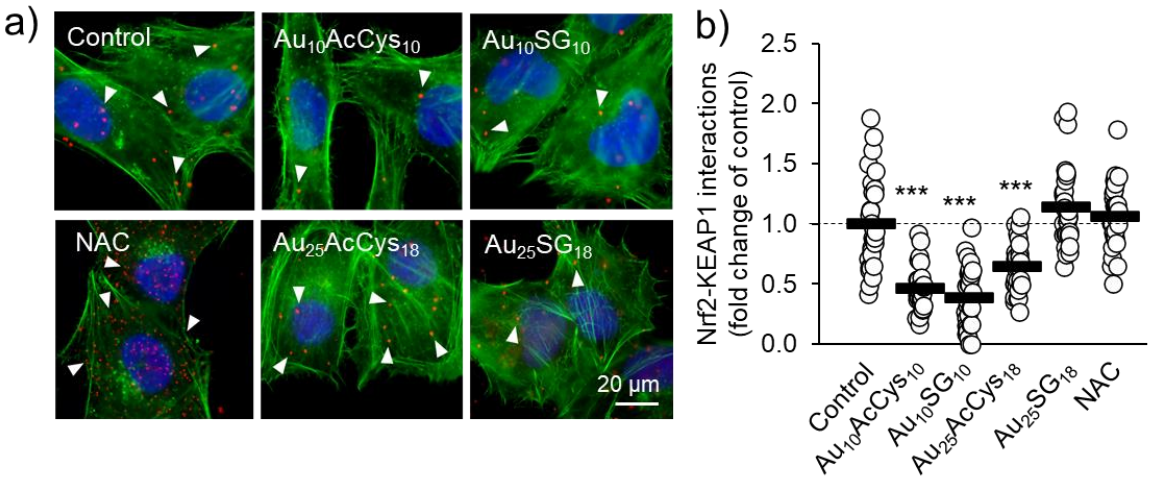

Supplementary Figure 8. Nrf2-KEAP1 interactions in human microglia treated with gold nanoclusters. a) Representative fluorescence micrographs of Nrf2-KEAP1 interactions (red dots, arrows) detected using proximity ligation assay. Cells were treated with $Au_{10}AcCys_{10}$, $Au_{10}SG_{10}$, $Au_{25}AcCys_{18}$ or $Au_{25}SG_{18}$ at 10 µM for 24h in serum-deprived conditions. Actin (green) is labelled with Alexa Fluor 488 Phalloidin. Nuclei (blue) are labeled with Hoechst 33342. **b)** Shown are the number of Nrf2-KEAP1 interactions per individual cell (white dot) in microglia treated as in **a)**, and the average per condition (black bar ±SD), as fold change of the untreated control (set to 1), from at least 120 cells and at least three independent experiments. NAC, N-acetyl-cysteine. ***$p<0.001$.

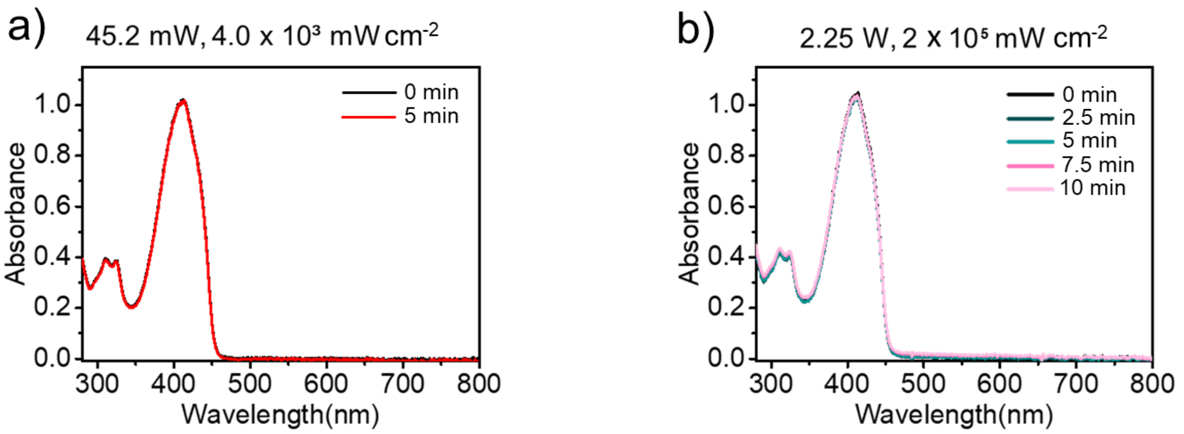

Supplementary Figure 9. Photostability of 1,3-diphenylisobenzofuran (DPBF) under 780 nm irradiation. Absorption spectra of DPBD dye under femtosecond laser irradiation (780 nm) at different laser power after several time points under irradiation, **a)** 4.0×10^3 mW cm^{-2}, **b)** 2×10^5 mW cm^{-2}.

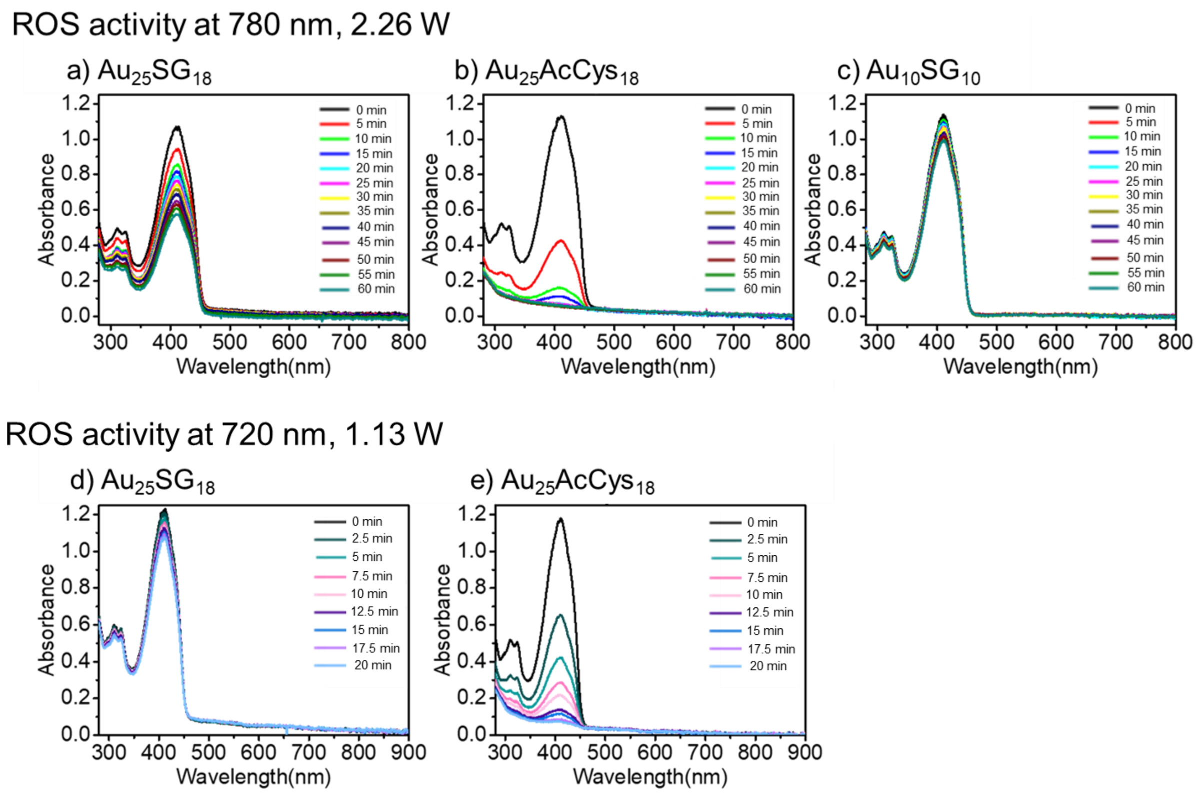

Supplementary Figure 10. Two-photon-excited singlet oxygen generation in solution of gold nanoclusters detected by 1,3-diphenylisobenzofuran (DPBF). Evolution of DPBF absorbance spectra in function of time under femtosecond laser (780 nm and 720 nm) irradiation with gold nanoclusters. **a)** DPBF with $Au_{25}SG_{18}$ under 780 nm, 4.0×10^3 mW cm^{-2}, **b)** DPBF with $Au_{25}AcCys_{18}$ under 780 nm, 4.0×10^3 mW cm^{-2}, **c)** DPBF with $Au_{10}SG_{10}$ under 780 nm, 4.0×10^3 mW cm^{-2}, **d)** DPBF with $Au_{25}SG_{18}$ under 720 nm, 2.0×10^3 mW cm^{-2}, **e)** DPBF with $Au_{25}AcCys_{18}$ under 780 nm, 4.0×10^3 mW cm^{-2}.

Supplementary Table 2. Singlet oxygen generation rate of metal nanoclusters under pulse 780 nm and 720 nm irradiation.

Photosensitizer	$^1O_2$ generation rate ($^1O_2$ per cluster per min) @780 nm, 4.0 × 10^3 mW cm^{-2}	$^1O_2$ generation rate ($^1O_2$ per cluster per min) @720 nm, 2.0 × 10^3 mW cm^{-2}	Two-photon absorption cross section
$Au_{10}SG_{10}$	0.1095		(800 nm) 10 GM [1]
$Au_{25}SG_{18}$	0.6994	0.3021	(800 nm) 189740 GM [2]
Au_{25}AcCys18	2.6958	2.6907	

Supplementary Table 3. Time-dependent density functional theory energies of singlet and triplet states in the different systems.

Excited states energies	$Au_{10}AcCys_{10}$	$Au_{10}SG_{10}$	$Au_{25}AcCys_{18}$	$Au_{25}SG_{18}$
	eV	eV	eV	eV
S_1	3.3615	3.2194	1.7298	1.7825
S_2	3.5413	3.4222	1.802	1.9257
S_3	3.9002	3.8222	2.002	1.9362
S_4	4.0445	3.9396	2.0192	2.0299
S_5	4.1036	3.9731	2.0863	2.1533
T_1	2.6797	2.5501	1.5841	1.6510
T_2	2.9797	2.8738	1.6222	1.7192
T_3	3.4058	3.1856	1.7653	1.7699
T_4	3.6418	3.5837	1.8378	1.8460
T_5	3.7067	3.6274	1.8915	1.9296
ΔEST	0.68	0.67	0.15	0.13

Supplementary References

1. Bertorelle, F. *et al.* Au10(SG)10: A Chiral Gold Catenane Nanocluster with Zero Confined Electrons. Optical Properties and First-Principles Theoretical Analysis. *J. Phys. Chem. Lett.* **8**, 1979–1985 (2017).
2. Polavarapu, L., Manna, M. & Xu, Q.-H. Biocompatible glutathione capped gold clusters as one- and two-photon excitation fluorescence contrast agents for live cells imaging. *Nanoscale* **3**, 429–434 (2011).
